# Genome integration of human DNA oncoviruses

**DOI:** 10.1128/jvi.00562-25

**Published:** 2025-07-23

**Authors:** Zuzana Vojtechova, Ruth Tachezy

**Affiliations:** 1 Department of Genetics and Microbiology, Faculty of Science BIOCEV, Charles University 37740, Prague, Czech Republic; Indiana University Bloomington, Bloomington, Indiana, USA

**Keywords:** virus, integration, hepatitis B virus, Epstein-Barr virus, human papillomavirus

## Abstract

Tumors of infectious origin globally represent 13%. Oncogenic DNA viruses such as human papillomavirus (HPV), hepatitis B virus (HBV), and Epstein-Barr virus (EBV) are responsible for approximately 60% of these tumors. These oncoviruses are extensively studied to understand their role in cancer development, particularly through viral genome integration into the host DNA. Retroviruses require integration mediated by viral integrase for persistence, whereas DNA oncoviruses do not need integration for replication; instead, integration occurs incidentally. This process often targets fragile sites in the human genome, causing structural rearrangements that disrupt genes, activate proto-oncogenes, and increase genomic instability, all contributing to tumorigenesis. Integration near promoter regions and active genes is closely linked to carcinogenesis, highlighting its importance in developing diagnostic and therapeutic strategies. This review summarizes viral integration’s role in oncogenesis, mechanisms of integration, and methods to study this process, focusing on DNA tumor viruses such as HBV, EBV, HPV, and Merkel cell polyomavirus.

## INTRODUCTION

More than 13% of global tumor malignancies are linked to pathogens (1), with *Helicobacter pylori* being the leading cause, responsible for over 35% of these cases. Nearly 60% of tumors of infectious origin are associated with DNA tumor viruses like human papillomavirus (HPV), hepatitis B virus (HBV), and Epstein-Barr virus (EBV) (1). Oncoviruses are extensively studied to understand their mechanisms in malignancy development.

For some viruses, such as retroviruses, integration of the viral genome into the host genome is essential and facilitated by the enzyme integrase, enabling lifelong persistence. Retroviral integration has been well-studied since 1990 (2–5). In contrast, DNA oncoviruses do not require integration for replication; it occurs incidentally and can disrupt genes, rearrange chromosomes, and promote oncogenesis. Despite significant research, many questions about viral integration remain, necessitating further study.

Viral integration into the human genome often targets fragile sites, causing structural rearrangements in viral DNA and host chromosomes. This process can alter gene expression, activate proto-oncogenes, and increase genomic instability, contributing to tumorigenesis. Integration near promoter regions and transcriptionally active genes is closely linked to carcinogenesis, highlighting its importance in developing diagnostic and therapeutic strategies for virus-associated cancers.

A unique case is human herpesvirus 6 (HHV-6), which integrates into the telomeric regions of germline cells and can be inherited as chromosomally integrated HHV-6 (iciHHV-6). IciHHV-6 has been identified as a risk factor for angina pectoris and acute graft-versus-host disease in hematopoietic cell transplant donor-recipient pairs (6, 7), though no statistically significant link to oncogenesis has been found (8).

This review will summarize the role of viral integration in oncogenesis, mechanisms of integration into the host genome, and methods for studying this process, focusing on DNA tumor viruses such as HBV, EBV, HPV, and Merkel cell polyomavirus (MCPyV).

## HEPATITIS B VIRUS

Human HBV, a small DNA virus in the *Hepadnaviridae* family, infects the liver and causes viral hepatitis. Despite the introduction of effective vaccination in the 1980s, 1.2 million new hepatitis B cases occur annually (9). HBV is transmitted via infectious blood or body fluids, and vertically from mother to child. Perinatal or childhood infection significantly increases the risk of chronic infection to 90% and 16–30%, respectively (10). While most adult infections resolve spontaneously, 5–10% become chronic, leading to cirrhosis or hepatocellular carcinoma (HCC) (11). HCC is the sixth most common cancer globally with over 900,000 new cases and 830,000 deaths annually (12). HBV accounts for approximately 46% of HCC cases and 56% of liver cancer deaths worldwide (12, 13).

The HBV genome consists of a partially double-stranded circular DNA (3.2 kbp) with an incomplete (+) strand. It encodes four overlapping open reading frames: preS1/preS2/S for surface proteins, preC/C for core protein and E antigen, P for polymerase, and X for HBx protein. The virus enters hepatocytes via the sodium taurocholate co-transporting polypeptide receptor, interacting with the preS1 domain of its large surface protein (14). After entry, the nucleocapsid is directed to the nucleus where relaxed circular DNA (rcDNA) is released. Host factors convert it into covalently closed circular DNA, serving as a template for viral RNA transcription (15–17). Reverse transcription of pre-genomic RNA produces new rcDNA or double-stranded linear DNA, which can integrate into the host genome but does not produce infectious particles (18, 19). Integration occurs at a frequency of ~1 per 10³–10⁴ hepatocytes *in vitro* (20).

HBV integration was first identified in the 1980s (21, 22) and is now recognized as a non-essential but early event in infection. It is detected in up to 90% of cirrhosis or HCC cases, but rarely in acute hepatitis patients (23–27). Integration contributes to HCC development by altering host gene expression, promoting chromosomal instability, and deregulating immune responses (28–30). The HBx protein plays a key role by modulating signaling pathways such as mitogen-activated protein kinase, nuclear factor κB (NF-κB), and Janus kinase/signal transducers and activators of transcription pathway to promote carcinogenesis (31, 32).

HBV integration frequently targets cancer-associated genes like human telomerase gene (*TERT*), the lysin methyltransferase 2B (*KMT2B*), cyclin E1 (*CCNE1*), and cyclin A2 (*CCNA2*), fragile genomic sites, and repetitive sequences, enhancing HCC risk (26, 27, 33–37). Chromosomal rearrangements associated with integration often involve gains at chr8q or chr5p or losses at chr17p affecting proto-oncogenes like *MYC* or tumor suppressors like TP53 (26). However, many integrations are passenger events without functional consequences. The distribution of integrated HBV on chromosomes is random in both tumor and non-malignant tissues.

Integrated HBV sequences often encode C-terminally truncated HBx proteins that disrupt cellular processes by activating signaling pathways or binding tumor suppressors like TP53, promoting tumorigenesis through angiogenesis, immune evasion, inflammation, and altered metabolism (32, 38–40). Integration is clonal in >48% of tumor cells. In adjacent non-tumor tissues, the integration occurs less frequently (<31%) (26, 36). In contrast, Péneau et al. identified HBV integration breakpoints in 84% of non-tumor tissues of patients with HCC, and most integration breakpoints in normal tissues were unique events (<3% of cells shared the same integration breakpoint) (26). Altogether, non-malignant and tumor tissues are concordant in less than 5% of samples (37, 41, 42).

Integration seems to impact clinicopathological features such as disease severity and prognosis (43). Tumors with high HBV integration levels are associated with poor survival outcomes independent of other factors like tumor size or differentiation (26, 36). Cell-free virus-host chimeric DNA detected postoperatively correlates with recurrence risk in HCC patients, highlighting its potential as a biomarker (44).

HBV integration confers resistance to antiviral therapy but provides stable targets for therapeutic intervention or prognostic biomarkers. Gene therapies targeting integration-derived transcripts offer promising strategies for managing HBV-related diseases (45).

## EPSTEIN-BARR VIRUS

EBV (human herpesvirus 4) is a human DNA oncovirus from the family *Herpesviridae*, genus *Gammaherpesvirinae*. Discovered in Burkitt’s lymphoma (BL) cells in 1964 (46), it is linked to 1.5% of all malignancies globally. EBV infects over 90% of adults early in life and typically enters a latent state after primary infection with potential for reactivation. While primary infection is often asymptomatic, it can manifest as infectious mononucleosis in young adults (47). EBV is associated with various epithelial and B cell-derived malignancies, including nasopharyngeal carcinoma (NPC) (48), BL (49), NK/T cell lymphoma (NKTCL) (50), Hodgkin’s lymphoma (HL) (51), and gastric cancer (52). According to Globocan, EBV ranks as the fifth most common cancer-related infection (1) causing over 160,000 deaths annually (53). Its prevalence in BL varies geographically from 20% in low-incidence areas to 95% in endemic regions (53). EBV is linked to 75–100% of NPC cases depending on region (54–56), 25–75% of NKTCL cases (54, 57), 31% of HL cases (57), and about 10% of gastric carcinomas (55). However, only a small fraction of infections result in malignancy, with factors like immunodeficiency, genetic predisposition, and environmental influences playing roles. EBV is also implicated in non-malignant diseases such as multiple sclerosis and oral hairy leukoplakia (58, 59).

EBV is an enveloped virus with a linear double-stranded DNA genome of 170–180 kbp encoding over 85 proteins and more than 40 non-coding RNAs (60–63). Transmitted via saliva, it infects the pharyngeal epithelium and resting B cells in underlying tissues (64). Unlike most enveloped viruses that use one or two glycoproteins for adhesion, EBV employs multiple glycoproteins like gp350/220, gH, gp42, and BMRF2 for cell entry (64, 65). Entry mechanisms differ between cell types: endocytosis for B cells and direct membrane fusion for epithelial cells (66). Once inside the host cell, the nucleocapsid travels to the nucleus where the viral genome circularizes (64, 67, 68). During the lytic cycle, immediate early genes are expressed first, followed by early genes for DNA replication and late genes for capsid and envelope proteins, culminating in mature virion release.

After primary infection, EBV can enter latency in B cells without producing viral particles but with altered gene expression. The viral genome persists as a circular episome during latency, with latent genes expressed alongside host DNA. Latency types (0, I, II, III) depend on the differentiation state of infected B cells (63). Latent genes include those encoding EBV nuclear antigens, latent membrane proteins, and non-coding RNAs like Epstein-Barr virus-encoded small RNAs (EBERs), microRNAs, or BamHI fragment A rightward transcripts. Reactivation from latency occurs under conditions such as T cell immunity attenuation, switching the virus back to the lytic cycle (69–71).

The carcinogenic mechanisms in EBV-associated tumors are primarily driven by viral latent gene products and non-coding RNAs, which promote cell proliferation, DNA damage, genomic instability, and chronic inflammation, all of which support tumor progression (72–76). While the latent state of EBV infection increases tumor development risk, evidence also suggests that lytic replication contributes to genetic instability and tumorigenesis (77).

During latency, the EBV genome persists as an episome but can also integrate into the host genome (78–81). Since the first reports in the 1980s (82–84), EBV integration has been confirmed in lymphomas and carcinomas, though its role in carcinogenesis remains incompletely understood. EBV integrates at a lower rate than other DNA viruses like HBV or HPV. Xu et al. found the highest integration rates in gastric carcinoma (26%) (85), followed by HL and NKTCL (18% and 16%, respectively). Late-stage NPC tumors and large gastric cancers showed increased integration rates, while Ohshima et al. identified integration in 11% of various tumor samples (79). Coexistence of episomal and integrated viral DNA has been observed in NPC, NKTCL, and BL cell lines (79, 81, 86). Hurley et al. reported higher integration frequency than the presence of an episomal form of the virus in persistently infected activated B cells (78).

Challenges in studying EBV integration include methylated DNA, interference from episomes, and the large viral genome size (87–89). However, studies have revealed key mechanisms linking integration to carcinogenesis. In BL-derived cell lines, viral integration near oncogenes like *REL* and *BCL11A* or tumor suppressor genes like *BACH2* alters gene expression or disrupts tumor suppressor functions, promoting proliferation and lymphomagenesis (88, 90). In NKTCL samples, integration sites were enriched in repetitive regions such as short interspersed nuclear elements (SINEs) and long interspersed nuclear elements (LINEs), forming chimeric transcripts that disrupt DNA repair or increase genome instability (81). Genome-wide profiling revealed integration near fragile sites or microsatellite repeats linked to DNA damage, affecting tumor suppressor genes like *KANK1*, *RB1CC1*, *PTEN*, *FHIT*, and *DLEC1* while dysregulating inflammatory pathways such as NF-κB or tumor necrosis factor alpha (TNF-alpha) apoptosis (85). EBV integration near fragile sites was observed in approximately one-third of cases (91, 92), with non-random distribution favouring certain chromosomal bands (91, 93). Chakravorty et al.’s interactome map showed preferential integration near highly expressed genes with super-enhancer regions and ribosomal RNA genes, significantly influencing host gene expression (54). Conversely, Xiao et al.’s findings suggested random integration correlated with structural variations in the host genome that increase instability (80).

EBV breakpoints are distributed throughout its genome with hotspots near *oriP* and terminal repeats. The identified microhomologies and insertions near integration sites suggest involvement of mediated repair pathways during integration (85). The integration of a full-length viral genome remains unclear due to read-length limitations in next-generation sequencing methods (85).

Like other viruses, EBV genome integration promotes carcinogenesis through multiple mechanisms, though its impact on patient prognosis remains unexplored. Interestingly, patients with EBV-associated malignancies tend to have better outcomes than those with EBV-negative tumors (94–96).

## HUMAN PAPILLOMAVIRUS

HPVs are small DNA tumor viruses of the *Papillomaviridae* family that infect epithelial cells. Over 450 HPV types have been identified, with 226 genomes cloned and stored in the International HPV Reference Center (97). Low-risk types cause benign growths like warts, while high-risk (HR) oncogenic types are crucial in the development of premalignant lesions, cervical cancer (CC), and other HPV-associated squamous cell carcinomas in the anogenital, head, and neck regions. HPV is the most common sexually transmitted viral infection globally, affecting both men and women, primarily through sexual or skin-to-skin contact. Vertical transmission from mother to child is rare (98). Studies have focused on women due to HPV’s high affinity for cervical cells. The WHO estimates a global HPV prevalence of 12% among women, peaking at 24% in sub-Saharan Africa (99, 100). A meta-analysis by Bruni et al. (101) revealed a global HR-HPV prevalence of 21% among men, highlighting the need for inclusive prevention strategies.

Most HPV infections are asymptomatic and resolve spontaneously. About 25% of infected individuals develop precancerous lesions, with less than 1% progressing to invasive cancer (102). HPV accounts for approximately 5% of all cancers worldwide, with over 690,000 new cases annually (1). Cervical cancer is nearly entirely caused by HPV and ranks as the fourth most common cancer and cause of cancer-related death in women, with an estimated 604,000 new cases and 342,000 deaths in 2020 (12). It is also the second most common cancer among women aged 15–44 (103). HPVs contribute to other anogenital cancers, such as those of the vagina, vulva, anus, and penis, with a combined incidence of 150,000 cases annually worldwide—nearly half attributable to HPV (1, 12). HR-HPV also plays a major role in head and neck cancers (HNC), particularly oropharyngeal squamous cell carcinoma (OPSCC), which affects the tonsils and tongue base. HNC is the eighth most common malignancy globally, with over 850,000 new cases annually (12). Approximately 30% of OPSCC cases are linked to HPV infection (104, 105), though proportions vary by region.

The HPV genome consists of ~8 kbp circular double-stranded DNA organized into three regions: the early region (E), encoding proteins essential for viral replication; the late region (L), encoding capsid proteins L1 and L2; and the non-coding long control region (LCR) containing regulatory sequences like replication origins and transcription factor binding sites (106). The viral life cycle depends on keratinocyte differentiation and host cell machinery. HPVs infect basal epithelial layers via microtraumas, with entry mediated by interactions between capsid protein L1 and heparan sulfate proteoglycans on basal cell membranes (107). In basal cells, the viral genome persists as an episome at low copy numbers but replicates extensively during epithelial differentiation. Capsid proteins are expressed during terminal differentiation, leading to genome encapsidation and virion release (108).

Persistent HR-HPV infection is essential for the development of premalignant lesions and their progression to invasive carcinoma (109). Low-grade cervical lesions (cervical intraepithelial lesion [CIN1]) are often transient and resolve within months (110). HPV oncogenes drive the progression to high-grade lesions (CIN2, CIN3) (111, 112), with E6 and E7 being key oncoproteins involved in cancer hallmarks (113). E6 forms a complex with E6AP and p53, leading to p53 degradation and loss of its tumor-suppressor functions (114–116). It also disrupts p53 binding to CBP/p300, activates telomerase, degrades PDZ-domain proteins, and modulates immune responses via IRF3 (117–121). E7 targets pRb for degradation, releasing E2F to promote cell cycle progression and interacting with other proteins to influence immune responses and cell regulation (122–126). E5 complements E6/E7 by enhancing EGFR signaling, inhibiting apoptosis, and promoting transformation (127–132).

Although most HPV infections are transient and asymptomatic, persistent infections can evade immune responses, enabling progression to carcinoma due to limited antigen production and reduced inflammation (133). Viral replication requires host DNA damage repair factors, but dysregulation of these pathways increases genomic instability and mutation rates through mechanisms such as reactive oxygen species (ROS) elevation by the E6* variant (134–138). HPV oncoproteins also induce centrosome accumulation, leading to aneuploidy (139–141).

Viral DNA integration into the host genome is considered an important event in HPV-associated carcinogenesis. While HPV initially exists as an extrachromosomal episome, integration frequency rises with disease severity (142, 143). Integrated forms are common in CCs (~80%) and head-and-neck squamous cell carcinomas (50–70%) (144–148). Integration disrupts regulatory genes like E2/E1, upregulating E6/E7 expression, while episomes may increase oncogene expression via copy number amplification or structural rearrangements (149, 150). Epigenetic modifications like DNA methylation may also enhance oncogene expression, though findings are inconsistent (150–153). Non-integrative carcinogenesis involving increased E2/E4/E5 expression has been proposed for certain cancers, suggesting alternative mechanisms for tumorigenesis (154).

Integration of HPV DNA into the host genome requires both viral and host DNA breakage, with the rate of integration influenced by factors such as inflammation, reactive oxygen/nitrogen species, environmental toxins, and apolipoprotein B mRNA editing enzyme (APOBEC) polypeptides (155–158). HPV integration occurs across almost all human chromosomes but is recurrently observed at sites like *PDL1*, *MYC*, *MACROD2*, and *KLF5* (145, 148, 159–163). These sites are often located near common fragile sites (CFSs), which are prone to chromosome breakage (164–166). Studies report HPV integration in CFSs in 38% of HPV-associated tumors and cell lines (167), consistent with findings in cervical carcinomas and tonsillar tumors (145, 168).

HPV integration can occur in intergenic regions or coding regions, often affecting cancer-associated genes such as tumor suppressors or oncogenes. Akagi et al. mapped breakpoints to intergenic loci (30%), exons (3%), introns (40%), and gene ends (28%) (169). Zhao et al. also observed a preference for intronic and intergenic regions (170). Integration can disrupt key genes like *RAD51B*, leading to loss of function and structural rearrangements that alter gene expression, such as *TP63* amplification or *MYC* overexpression via the viral promoter (171). Fusion transcripts driven by the viral LCR are highly expressed at productive integration sites, as shown in CC studies (162). In OPSCC, integration affects tumor-related pathways in nearly half of cases analyzed (172). Tian et al. identified HPV host extrachromosomal DNA containing super-enhancers that dysregulate chromosomal gene expression, proposing novel oncogenic functions for extrachromosomal DNA reservoirs (173).

Epigenetic changes also play a role in HPV integration’s oncogenic effects. Methylation analyses suggest that integrated HPV genomes share methylation patterns with flanking human sequences, impacting gene expression (174). Tandem integrations may involve silenced complete viral copies, while incomplete sequences at breakpoints affect host genes (175, 176). Zeng et al. found that HPV integration into enhancer regions, such as MIR205HG, reduces methylation and upregulates expression, contributing to carcinogenesis (163).

The disruption of the viral E2 gene during integration is thought to weaken E2-mediated regulation of oncogene expression, but studies suggest that constitutive rather than high-level oncogene expression is sufficient for oncogenesis (177). Deletions in other viral genes like *E1*, *E5*, *L1*, or *L2* have also been observed upon integration (148), with breakpoints often occurring in *L1*/*L2* rather than *E2* regions in CC (170).

The prognostic significance of HPV integration remains unclear due to methodological differences and small cohorts studied across HNC and CCs. Several studies reported no significant difference in disease-specific survival between patients with integrated vs. episomal or mixed forms of HPV but noted trends favoring mixed forms for longer recurrence-free survival (145, 178, 179). Contrastingly, Koneva et al., using TCGA data, found poor survival associated with integration, particularly in older patients (180, 181). Conversely, Pinatti et al.’s recent findings suggest HPV integration could serve as a marker of good prognosis (182).

In summary, while increased expression of viral oncogenes is a key mechanism in HPV-driven oncogenesis, genomic alterations, changes in the genome that result from the integration of the virus, contribute significantly to this multifaceted process through structural rearrangements, epigenetic changes, and dysregulated gene expression pathways.

## MERKEL CELL POLYOMAVIRUS

MCPyV is a small, non-enveloped, double-stranded DNA virus first identified in 2008 (183). It belongs to the *Polyomaviridae* family and is the only polyomavirus linked to human tumorigenesis. MCPyV is found in 80% of Merkel cell carcinomas (MCC), a rare but aggressive skin cancer (184). The cellular origin of MCC remains controversial, with viral and non-viral MCC likely arising from different skin cell types (185). MCC incidence is approximately three per million globally, with geographical variations (186). In the U.S., MCC incidence rose 3.5-fold from 1991 to 2016, continuing to increase with age due to immune senescence (187). The highest rates are in Australia, at 3.9 per 100,000 men and 2.5 per 100,000 women annually (188). Non-viral MCC is linked to UV-induced mutations, while viral MCC involves MCPyV infection and virus-induced carcinogenesis.

MCPyV has also been detected in non-MCC skin lesions such as melanoma (4%), squamous cell carcinoma (15%), basal cell carcinoma (14%), and actinic keratosis (6%), as well as in normal skin, with prevalence ranging from 11-68% across studies (184, 189–192). It has also been found in 10% of non-malignant tonsillar tissues (193). Seroprevalence data suggest MCPyV is widespread, with initial infections occurring in childhood and increasing with age (194–196).

The MCPyV genome is a circular, double-stranded DNA of 5.4 kbp comprising two transcription units and a non-coding control region. The E encodes small and large T antigens (sT and LT), a splice variant (57kT), an alternative LT open reading frame, and a viral miRNA (MCV miR M1) (183, 197, 198). The L encodes structural proteins VP1 and VP2 but lacks VP3, unlike other polyomaviruses (199, 200). Viral attachment involves VP1 binding to sulfated glycosaminoglycans and sialic acid-containing co-receptors, followed by transport through endosomes to the nucleus via nucleopores (201–203). Viral gene expression is restricted to human dermal fibroblasts, with early genes initiating replication and late genes regulating capsid protein expression for virion assembly and release (204–206).

MCPyV-associated MCC development requires two key mutagenic events: truncation of the LT antigen sequence and viral genome integration into the host genome (183, 207, 208). LT truncation impairs helicase activity but preserves pRb-binding ability, disrupting tumor suppressor function and promoting oncogenesis (209). sT antigen expression remains intact, contributing to oncogenesis by inhibiting proteasomal degradation of oncoproteins like cyclin E or c-Myc (210–212). Viral integration occurs randomly in the host genome, often near repeat elements like SINEs or LINEs, with chromosome 5 being a frequent site (213–215). Integration disrupts LT gene sequences downstream of the pRb-binding site while preserving its oncogenic functions.

Integration often occurs as single copies or tandem concatemers by the mechanism of rolling circle amplification without cleavage of unit-length genomes (215, 216). Most integration sites are intronic or intergenic regions, occasionally affecting genes directly involved in carcinogenesis (213, 217). Integrated MCPyV genomes frequently undergo large deletions, with over one-third of tumors showing deletions of at least half the viral genome (214). Integration is an early event in MCC carcinogenesis, as primary tumors and metastases often share integration patterns.

Patients with MCPyV-positive MCC generally have better prognoses than those with non-viral MCCs, though the impact of integration on survival remains underexplored due to the rarity of MCPyV-associated tumors with extensive genomic changes (218, 219).

## MECHANISMS OF DNA TUMOR VIRUS INTEGRATION

**Fig 1:**
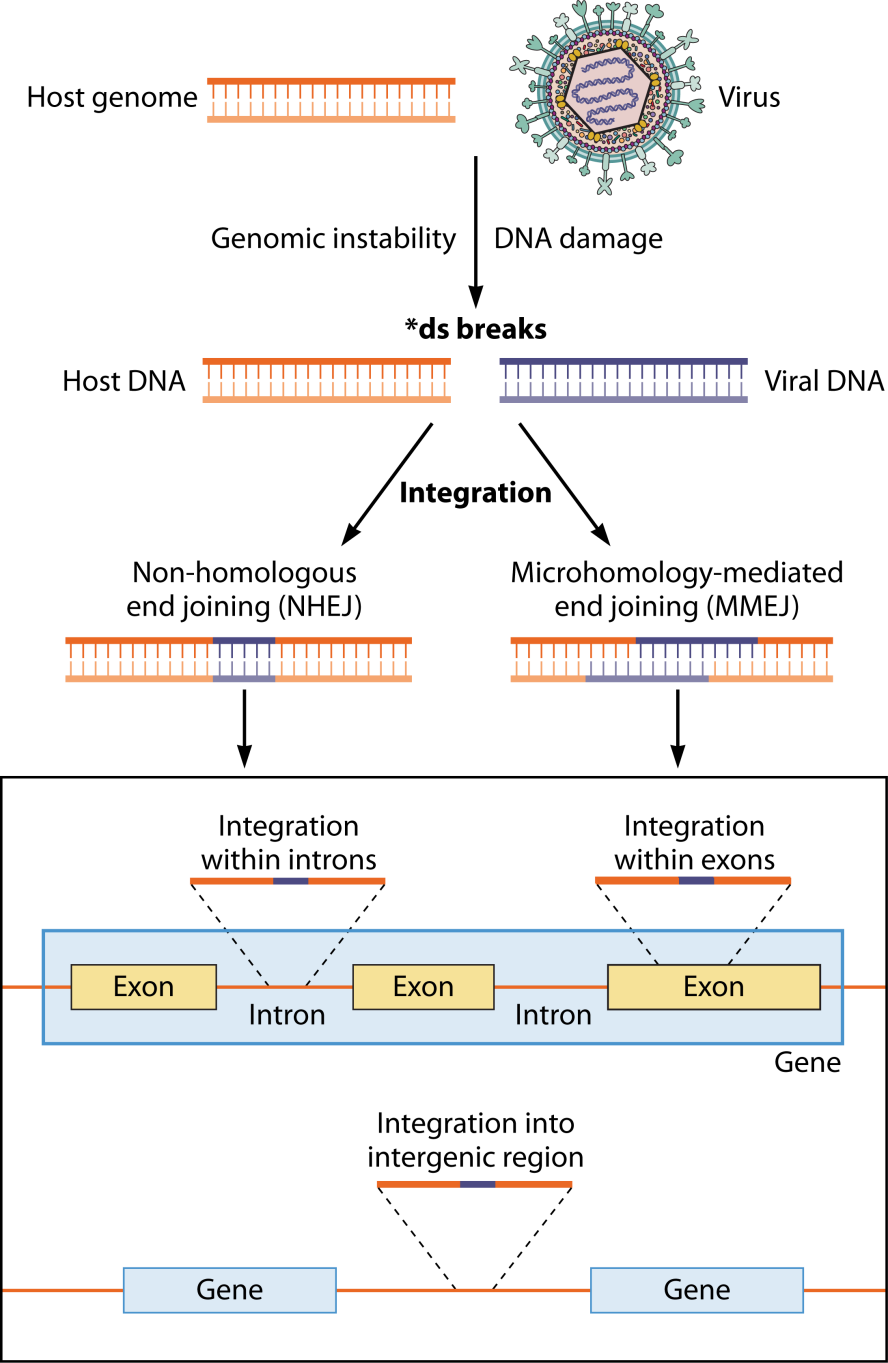
Diagram illustrating the main mechanisms of viral integration and the host genome regions that can be affected by viral integration, resulting in changes in gene expression. Created with BioRender.com.

Viral DNA integration into the host genome primarily occurs through the repair of DNA double-stranded breaks (DSBs) at sites of genomic instability or damage, mainly via non-homologous end joining (NHEJ) without sequence homology between virus and host DNA. A secondary mechanism involves microhomology-mediated end joining (MMEJ) (Fig. 1). Both processes are observed in HBV integration (20, 220). Integrated viral DNA fragments range from 28 bp to the full-length HBV sequence (221). Around 37–40% of HBV breakpoints map between short repetitive sequences DR1 and DR2 (41, 222). In EBV, sequencing and computational analysis revealed that integration is primarily mediated by canonical NHEJ, followed by synthesis-dependent end joining, and other alternative mechanisms like MMEJ or replication fork stalling and template switching during DSB repair (223).

For HPV, integration mechanisms remain less understood, but two models have been proposed. The “looping model” suggests HPV DNA forms a loop between two host DNA fragments with DSBs, leading to amplification, rearrangement into concatemers, or excision into viral/host fusion episomes (169, 224–226). The second model involves direct integration via MMEJ, where HPV oncoproteins interfere with DNA damage repair pathways, leading to integration at microhomologous regions enriched near breakpoints (159). MMEJ is an error-prone backup when homologous repair (HR) fails to repair DSBs (227, 228). While HPV uses HR for replication, its role in integration remains unproven, and the switch from high-fidelity HR to error-prone MMEJ during integration is not yet elucidated (225).

**Fig 2:**
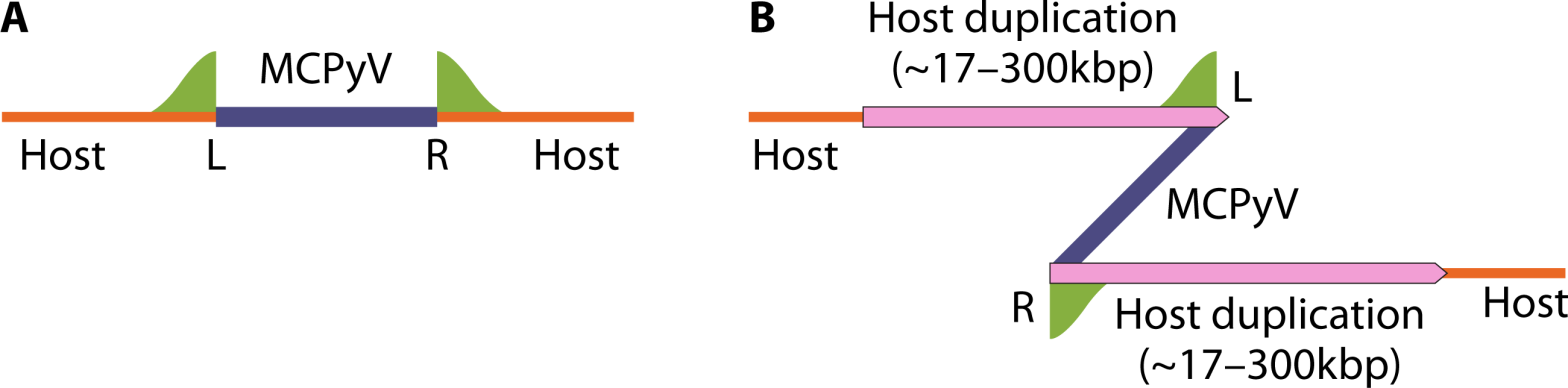
Patterns of MCPyV integration. (**A**) Linear integration pattern (L—left integration breakpoint and R—right integration breakpoint) and (**B**) Z-pattern integration. (Adapted from reference 215.)

Two integration patterns have been proposed for MCPyV: NHEJ, resulting in a linear integration pattern (Fig. 2A) with minor host DNA loss and MMEJ, creating a Z-pattern integration (Fig. 2B) with distant breakpoints and duplication of adjacent host sequences after rolling cycle amplification (215, 216). These events may also involve additional rearrangements or amplifications.

## METHODS USED FOR VIRAL INTEGRATION ANALYSIS

Many methodological approaches have been reported for viral integration analysis. Historically, Southern blot hybridization has been used (21, 229). While inexpensive, it is limited in detecting viral copies per cell and cannot determine the nucleotide sequence of the integration site. Southern blot remains a standard for determining HPV genome status, including concatemers, but it is time-consuming and requires large amounts of fresh DNA, restricting its use in large or retrospective studies (145, 230). For EBV integration, fractionation of DNA on a cesium cloride (CsCl) density gradient followed by a Southern blot has been employed (83). Electrophoresis-based methods, such as those described by Gardella et al., detect linear and episomal EBV DNA (231–233). Pulse-field gel electrophoresis further improved the separation of long DNA molecules for EBV analysis (87).

Southern blot analysis has been complemented by fluorescence *in situ* hybridization (FISH) (79, 232), which is now widely used. Hybridization-based methods like *in situ* hybridization (ISH) or FISH detect HPV integration at the single-cell level and determine whether integrated viral sequences are transcribed or silent (234–236). These methods are sensitive and applicable to fixed tissues but require prior knowledge of the sequence of interest for probe design and are relatively expensive compared to PCR-based techniques developed later. ISH with radiolabeled probes has also been used for HBV integrants' chromosomal positioning but with low sensitivity (237). Fiber FISH, combining molecular combing and FISH, characterizes HPV genomic integration sites containing tandemly integrated genomes interspersed with host DNA (238).

Direct cloning and Sanger sequencing were initially used to identify exact integration sequences but are technically demanding, low-throughput, and reliant on correct restriction enzymes (239). More sensitive PCR-based methods combined with sequencing now allow detailed resolution of host-cell genome integration (80, 81, 85). For HBV analysis, primers targeting Alu repetitive elements, which constitute over 10% of the human genome, were paired with HBV-specific primers (25, 240). However, this method is limited to detecting integrations near Alu sequences without quantifying junctions.

Inverse PCR detects single-copy HBV cell junctions and quantifies their absolute number but depends on restriction enzyme sites, limiting its scope (20, 241). DIPS PCR (detection of integrated papillomavirus sequences), developed for HPV analysis, maps chimeric virus-host DNA sequences through single-side ligation-mediated PCR followed by sequencing, enabling determination of HPV genome status and integration sites but requiring high-quality DNA and optimization due to reliance on restriction enzymes (242, 243). This method has also been applied to MCPyV integration in MCC (213, 217, 244).

The amplification of papilloma virus oncogene transcripts (APOT) assay is a highly sensitive RNA-based method using modified 3′ rapid amplification of cDNA ends (RACE) to analyze actively transcribed sequences but requires high-quality RNA (145, 172, 245, 246). It involves reverse transcription with adaptor-linked oligo dT primers followed by two PCR steps with adaptor primers and HPV-specific primers. Amplification products can be hybridized to HPV-specific probes, or the fusion transcripts can be sequenced to determine the exact integration breakpoint. Quantitative PCR, which measures the E2/E6 DNA ratio based on frequent E2 gene deletions during integration, is commonly used but cannot distinguish between tandem repeats and extrachromosomal forms of HPV (247–249).

Next-generation sequencing (NGS) has revolutionized viral integration detection by revealing exact sites with high sensitivity without prior sequence knowledge. NGS methods include whole-genome sequencing, whole-exome sequencing, RNA sequencing, capture-enriched NGS, or RAISING for HBV analysis (26, 27, 40, 215, 224, 250–255). Despite their sensitivity and applicability to diverse biological materials like formalin-fixed paraffin embedded (FFPE) tissue or blood, NGS methods remain costly and require complex bioinformatics analyses.

Nanopore sequencing provides long DNA reads with lower accuracy but reduces experimental artefacts due to unnecessary or less intense fragmentation of the DNA (256, 257). Nanochannel sequencing has recently been applied to MCPyV integration mapping single DNA molecules with rapid determination of copy numbers and adjacent host sequences' patterns (215).

For EBV integration, DeepEBV employs deep learning to predict integration sites based solely on DNA sequences, offering a promising tool for precise EBV research when combined with known results (258).

## CONCLUDING REMARKS

**Fig 3:**
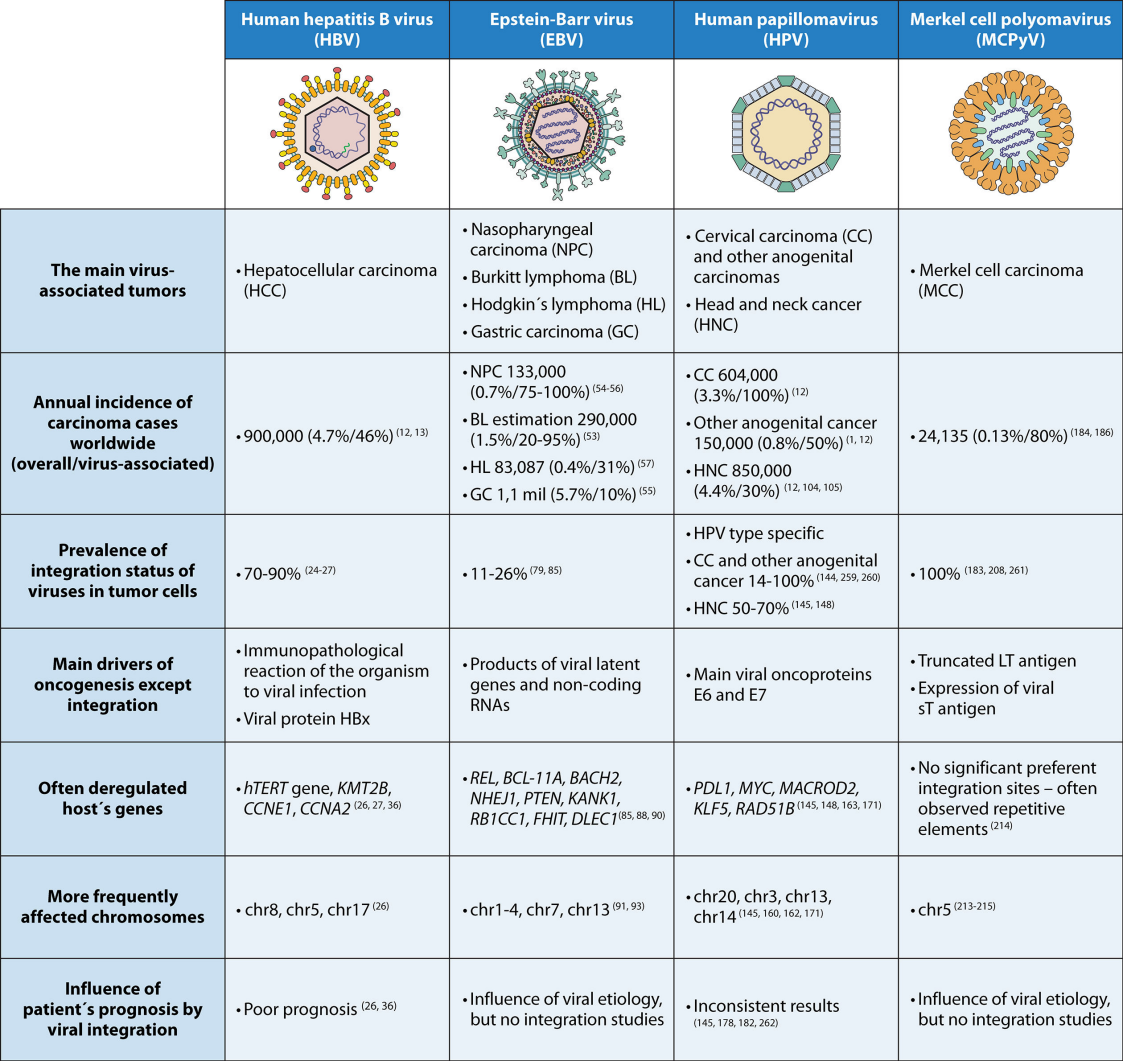
Comparison of the characteristics of particular DNA tumor viruses. Created with BioRender.com.

DNA oncoviruses are extensively studied to elucidate their oncogenic pathways and molecular effects. A key mechanism involves integration into the host genome, altering cellular pathways to promote cell cycle deregulation and tumorigenesis. This review highlights carcinogenesis linked to prevalent DNA tumor viruses and examines how genomic integration affects tumor development and prognosis (for summary see Fig. 3).

Viral DNA integration is critical in HBV-associated cirrhosis and HCC, where it occurs in over 70% of cases. Alongside chronic infection and inflammation, this process drives HBV progression to HCC. In contrast, EBV integrates less frequently, suggesting its oncogenic role arises from alternative mechanisms, such as modulation of cellular signaling by EBV-encoded proteins.

HPV integration is well-studied in cervical and HNC. HPV oncoproteins deregulate the cell cycle and apoptosis, leading to cellular transformation. Similarly, MCPyV integration is common in MCC, where it facilitates tumor initiation alongside LT mutations.

This review also explores viral genome integration mechanisms and the methods used to analyze them, emphasizing the advantages and limitations of these approaches. Advances in NGS provide sensitive tools for studying viral integration, despite challenges such as complex bioinformatics analyses. These methods promise to uncover previously unexplored data.

Despite progress, many questions remain. Future research on viral integration could identify new therapeutic targets to improve patient outcomes. However, focused studies on viral integration are needed before clinical application.

## References

[1] de Martel C, Georges D, Bray F, Ferlay J, Clifford GM. 2020. Global burden of cancer attributable to infections in 2018: a worldwide incidence analysis. Lancet Glob Health 8:e180–e190. doi:10.1016/S2214-109X(19)30488-7 31862245

[2] Grandgenett DP, Mumm SR. 1990. Unraveling retrovirus integration. Cell 60:3–4. doi:10.1016/0092-8674(90)90707-l 2403842

[3] Serrao E, Engelman AN. 2016. Sites of retroviral DNA integration: from basic research to clinical applications. Crit Rev Biochem Mol Biol 51:26–42. doi:10.3109/10409238.2015.1102859 26508664 PMC4866806

[4] Lesbats P, Engelman AN, Cherepanov P. 2016. Retroviral DNA integration. Chem Rev 116:12730–12757. doi:10.1021/acs.chemrev.6b00125 27198982 PMC5084067

[5] Maertens GN, Engelman AN, Cherepanov P. 2022. Structure and function of retroviral integrase. Nat Rev Microbiol 20:20–34. doi:10.1038/s41579-021-00586-9 34244677 PMC8671357

[6] Gravel A, Dubuc I, Morissette G, Sedlak RH, Jerome KR, Flamand L. 2015. Inherited chromosomally integrated human herpesvirus 6 as a predisposing risk factor for the development of angina pectoris. Proc Natl Acad Sci USA 112:8058–8063. doi:10.1073/pnas.1502741112 26080419 PMC4491735

[7] Hill JA, Magaret AS, Hall-Sedlak R, Mikhaylova A, Huang M-L, Sandmaier BM, Hansen JA, Jerome KR, Zerr DM, Boeckh M. 2017. Outcomes of hematopoietic cell transplantation using donors or recipients with inherited chromosomally integrated HHV-6. Blood 130:1062–1069. doi:10.1182/blood-2017-03-775759 28596425 PMC5570681

[8] Gravel A, Dubuc I, Brooks-Wilson A, Aronson KJ, Simard J, Velásquez-García HA, Spinelli JJ, Flamand L. 2017. Inherited chromosomally integrated human herpesvirus 6 and breast cancer. Cancer Epidemiol Biomarkers Prev 26:425–427. doi:10.1158/1055-9965.EPI-16-0735 27777240

[9] Global hepatitis report. 2024. Available from: https://www.who.int/publications/b/68511. Retrieved 15 Jul 2025.

[10] Pollicino T, Caminiti G. 2021. HBV-integration studies in the clinic: role in the natural history of infection. Viruses 13:368. doi:10.3390/v13030368 33652619 PMC7996909

[11] Shi Y-H, Shi C-H. 2009. Molecular characteristics and stages of chronic hepatitis B virus infection. World J Gastroenterol 15:3099–3105. doi:10.3748/wjg.15.3099 19575488 PMC2705731

[12] Sung H, Ferlay J, Siegel RL, Laversanne M, Soerjomataram I, Jemal A, Bray F. 2021. Global Cancer Statistics 2020: GLOBOCAN estimates of incidence and mortality worldwide for 36 cancers in 185 countries. CA Cancer J Clin 71:209–249. doi:10.3322/caac.21660 33538338

[13] Plummer M, de Martel C, Vignat J, Ferlay J, Bray F, Franceschi S. 2016. Global burden of cancers attributable to infections in 2012: a synthetic analysis. Lancet Glob Health 4:e609–e616. doi:10.1016/S2214-109X(16)30143-7 27470177

[14] Yan H, Zhong G, Xu G, He W, Jing Z, Gao Z, Huang Y, Qi Y, Peng B, Wang H, Fu L, Song M, Chen P, Gao W, Ren B, Sun Y, Cai T, Feng X, Sui J, Li W. 2012. Sodium taurocholate cotransporting polypeptide is a functional receptor for human hepatitis B and D virus. Elife 3:e00049. doi:10.7554/eLife.00049 PMC3485615 23150796

[15] Long Q, Yan R, Hu J, Cai D, Mitra B, Kim ES, Marchetti A, Zhang H, Wang S, Liu Y, Huang A, Guo H. 2017. The role of host DNA ligases in hepadnavirus covalently closed circular DNA formation. PLoS Pathog 13:e1006784. doi:10.1371/journal.ppat.1006784 29287110 PMC5747486

[16] Kitamura K, Que L, Shimadu M, Koura M, Ishihara Y, Wakae K, Nakamura T, Watashi K, Wakita T, Muramatsu M. 2018. Flap endonuclease 1 is involved in cccDNA formation in the hepatitis B virus. PLoS Pathog 14:e1007124. doi:10.1371/journal.ppat.1007124 29928064 PMC6013022

[17] Tang L, Sheraz M, McGrane M, Chang J, Guo J-T. 2019. DNA polymerase alpha is essential for intracellular amplification of hepatitis B virus covalently closed circular DNA. PLoS Pathog 15:e1007742. doi:10.1371/journal.ppat.1007742 31026293 PMC6505960

[18] Haines KM, Loeb DD. 2007. The sequence of the RNA primer and the DNA template influence the initiation of plus-strand DNA synthesis in hepatitis B virus. J Mol Biol 370:471–480. doi:10.1016/j.jmb.2007.04.057 17531265 PMC1991300

[19] Zhao X-L, Yang J-R, Lin S-Z, Ma H, Guo F, Yang R-F, Zhang H-H, Han J-C, Wei L, Pan X-B. 2016. Serum viral duplex-linear DNA proportion increases with the progression of liver disease in patients infected with HBV. Gut 65:502–511. doi:10.1136/gutjnl-2014-308989 26045139

[20] Tu T, Budzinska MA, Vondran FWR, Shackel NA, Urban S. 2018. Hepatitis B virus DNA integration occurs early in the viral life cycle in an in vitro infection model via sodium taurocholate cotransporting polypeptide-dependent uptake of enveloped virus particles. J Virol 92:e02007-17. doi:10.1128/JVI.02007-17 29437961 PMC5952132

[21] Brechot C, Pourcel C, Louise A, Rain B, Tiollais P. 1980. Presence of integrated hepatitis B virus DNA sequences in cellular DNA of human hepatocellular carcinoma. Nature 286:533–535. doi:10.1038/286533a0 6250074

[22] Shafritz DA, Shouval D, Sherman HI, Hadziyannis SJ, Kew MC. 1981. Integration of hepatitis B virus DNA into the genome of liver cells in chronic liver disease and hepatocellular carcinoma. Studies in percutaneous liver biopsies and post-mortem tissue specimens. N Engl J Med 305:1067–1073. doi:10.1056/NEJM198110293051807 6268980

[23] Kimbi GC, Kramvis A, Kew MC. 2005. Integration of hepatitis B virus DNA into chromosomal DNA during acute hepatitis B. World J Gastroenterol 11:6416–6421. doi:10.3748/wjg.v11.i41.6416 16425409 PMC4355779

[24] Bréchot C. 2004. Pathogenesis of hepatitis B virus-related hepatocellular carcinoma: old and new paradigms. Gastroenterology 127:S56–S61. doi:10.1053/j.gastro.2004.09.016 15508104

[25] Murakami Y, Saigo K, Takashima H, Minami M, Okanoue T, Bréchot C, Paterlini-Bréchot P. 2005. Large scaled analysis of hepatitis B virus (HBV) DNA integration in HBV related hepatocellular carcinomas. Gut 54:1162–1168. doi:10.1136/gut.2004.054452 16009689 PMC1774867

[26] Péneau C, Imbeaud S, La Bella T, Hirsch TZ, Caruso S, Calderaro J, Paradis V, Blanc J-F, Letouzé E, Nault J-C, Amaddeo G, Zucman-Rossi J. 2022. Hepatitis B virus integrations promote local and distant oncogenic driver alterations in hepatocellular carcinoma. Gut 71:616–626. doi:10.1136/gutjnl-2020-323153 33563643 PMC8862055

[27] Zhao L-H, Liu X, Yan H-X, Li W-Y, Zeng X, Yang Y, Zhao J, Liu S-P, Zhuang X-H, Lin C, et al.. 2016. Genomic and oncogenic preference of HBV integration in hepatocellular carcinoma. Nat Commun 7:12992. doi:10.1038/ncomms12992 27703150 PMC5059470

[28] Berasain C, Castillo J, Perugorria MJ, Latasa MU, Prieto J, Avila MA. 2009. Inflammation and liver cancer: new molecular links. Ann N Y Acad Sci 1155:206–221. doi:10.1111/j.1749-6632.2009.03704.x 19250206

[29] Chen Y, Tian Z. 2019. HBV-induced immune imbalance in the development of HCC. Front Immunol 10:2048. doi:10.3389/fimmu.2019.02048 31507621 PMC6718466

[30] Zhang HH, Mei MH, Fei R, Liu F, Wang JH, Liao WJ, Qin LL, Wei L, Chen HS. 2010. Regulatory T cells in chronic hepatitis B patients affect the immunopathogenesis of hepatocellular carcinoma by suppressing the anti-tumour immune responses. J Viral Hepat 17:34–43. doi:10.1111/j.1365-2893.2010.01269.x 20586932

[31] Kim CM, Koike K, Saito I, Miyamura T, Jay G. 1991. HBx gene of hepatitis B virus induces liver cancer in transgenic mice. Nature 351:317–320. doi:10.1038/351317a0 2034275

[32] Sivasudhan E, Blake N, Lu Z, Meng J, Rong R. 2022. Hepatitis B viral protein HBx and the molecular mechanisms modulating the hallmarks of hepatocellular carcinoma: a comprehensive review. Cells 11:741. doi:10.3390/cells11040741 35203390 PMC8870387

[33] Horikawa I, Barrett JC. 2001. cis-Activation of the human telomerase gene (hTERT) by the hepatitis B virus genome. J Natl Cancer Inst 93:1171–1173. doi:10.1093/jnci/93.15.1171 11481390

[34] Lin SY, Zhang A, Lian J, Wang J, Chang T-T, Lin Y-J, Song W, Su Y-H. 2021. Recurrent HBV integration targets as potential drivers in hepatocellular carcinoma. Cells 10:1294. doi:10.3390/cells10061294 34071075 PMC8224658

[35] Feitelson MA, Lee J. 2007. Hepatitis B virus integration, fragile sites, and hepatocarcinogenesis. Cancer Lett 252:157–170. doi:10.1016/j.canlet.2006.11.010 17188425

[36] Sung W-K, Zheng H, Li S, Chen R, Liu X, Li Y, Lee NP, Lee WH, Ariyaratne PN, Tennakoon C, et al.. 2012. Genome-wide survey of recurrent HBV integration in hepatocellular carcinoma. Nat Genet 44:765–769. doi:10.1038/ng.2295 22634754

[37] Jang J-W, Kim H-S, Kim J-S, Lee S-K, Han J-W, Sung P-S, Bae S-H, Choi J-Y, Yoon S-K, Han D-J, Kim T-M, Roberts LR. 2021. Distinct patterns of HBV integration and TERT alterations between in tumor and non-tumor tissue in patients with hepatocellular carcinoma. Int J Mol Sci 22:7056. doi:10.3390/ijms22137056 34209079 PMC8268258

[38] Schlüter V, Meyer M, Hofschneider PH, Koshy R, Caselmann WH. 1994. Integrated hepatitis B virus X and 3’ truncated preS/S sequences derived from human hepatomas encode functionally active transactivators. Oncogene 9:3335–3344. 7936659

[39] Liu X-H, Lin J, Zhang S-H, Zhang S-M, Feitelson M-A, Gao H-J, Zhu M-H. 2008. COOH-terminal deletion of HBx gene is a frequent event in HBV-associated hepatocellular carcinoma. World J Gastroenterol 14:1346. doi:10.3748/wjg.14.1346 18322946 PMC2693680

[40] Fujimoto A, Totoki Y, Abe T, Boroevich KA, Hosoda F, Nguyen HH, Aoki M, Hosono N, Kubo M, Miya F, et al.. 2012. Whole-genome sequencing of liver cancers identifies etiological influences on mutation patterns and recurrent mutations in chromatin regulators. Nat Genet 44:760–764. doi:10.1038/ng.2291 22634756

[41] Jiang S, Yang Z, Li W, Li X, Wang Y, Zhang J, Xu C, Chen P-J, Hou J, McCrae MA, Chen X, Zhuang H, Lu F. 2012. Re-evaluation of the carcinogenic significance of hepatitis B virus integration in hepatocarcinogenesis. PLoS One 7:e40363. doi:10.1371/journal.pone.0040363 22962577 PMC3433482

[42] Yang X, Wu L, Lin J, Wang A, Wan X, Wu Y, Robson SC, Sang X, Zhao H. 2017. Distinct hepatitis B virus integration patterns in hepatocellular carcinoma and adjacent normal liver tissue. Int J Cancer 140:1324–1330. doi:10.1002/ijc.30547 27943263

[43] Katoh H, Shibata T, Kokubu A, Ojima H, Loukopoulos P, Kanai Y, Kosuge T, Fukayama M, Kondo T, Sakamoto M, Hosoda F, Ohki M, Imoto I, Inazawa J, Hirohashi S. 2005. Genetic profile of hepatocellular carcinoma revealed by array-based comparative genomic hybridization: identification of genetic indicators to predict patient outcome. J Hepatol 43:863–874. doi:10.1016/j.jhep.2005.05.033 16139920

[44] Li C-L, Ho M-C, Lin Y-Y, Tzeng S-T, Chen Y-J, Pai H-Y, Wang Y-C, Chen C-L, Lee Y-H, Chen D-S, Yeh S-H, Chen P-J. 2020. Cell-free virus-host chimera DNA from hepatitis B virus integration sites as a circulating biomarker of hepatocellular cancer. Hepatology 72:2063–2076. doi:10.1002/hep.31230 32171027

[45] Salpini R, D’Anna S, Benedetti L, Piermatteo L, Gill U, Svicher V, Kennedy PTF. 2022. Hepatitis B virus DNA integration as a novel biomarker of hepatitis B virus-mediated pathogenetic properties and a barrier to the current strategies for hepatitis B virus cure. Front Microbiol 13:972687. doi:10.3389/fmicb.2022.972687 36118192 PMC9478028

[46] Epstein MA, Achong BG, Barr YM. 1964. Virus particles in cultured lymphoblasts from Burkitt’s lymphoma. Lancet 1:702–703. doi:10.1016/s0140-6736(64)91524-7 14107961

[47] Rostgaard K, Balfour HH, Jarrett R, Erikstrup C, Pedersen O, Ullum H, Nielsen LP, Voldstedlund M, Hjalgrim H. 2019. Primary Epstein-Barr virus infection with and without infectious mononucleosis. PLoS One 14:e0226436. doi:10.1371/journal.pone.0226436 31846480 PMC6917282

[48] Su ZY, Siak PY, Leong C-O, Cheah S-C. 2023. The role of Epstein-Barr virus in nasopharyngeal carcinoma. Front Microbiol 14:1116143. doi:10.3389/fmicb.2023.1116143 36846758 PMC9947861

[49] Campanero MR. 2008. Mechanisms involved in Burkitt’s tumor formation. Clin Transl Oncol 10:250–255. doi:10.1007/s12094-008-0193-x 18490240

[50] Allen PB, Lechowicz MJ. 2019. Management of NK/T-cell lymphoma, nasal type. J Oncol Pract 15:513–520. doi:10.1200/JOP.18.00719 31600461 PMC6790879

[51] Farrell K, Jarrett RF. 2011. The molecular pathogenesis of Hodgkin lymphoma. Histopathology 58:15–25. doi:10.1111/j.1365-2559.2010.03705.x 21261680

[52] Sun K, Jia K, Lv H, Wang S-Q, Wu Y, Lei H, Chen X. 2020. EBV-positive gastric cancer: current knowledge and future perspectives. Front Oncol 10:583463. doi:10.3389/fonc.2020.583463 33381453 PMC7769310

[53] Khan G, Fitzmaurice C, Naghavi M, Ahmed LA. 2020. Global and regional incidence, mortality and disability-adjusted life-years for Epstein-Barr virus-attributable malignancies, 1990-2017. BMJ Open 10:e037505. doi:10.1136/bmjopen-2020-037505 PMC7462312 32868361

[54] Chakravorty S, Yan B, Wang C, Wang L, Quaid JT, Lin CF, Briggs SD, Majumder J, Canaria DA, Chauss D, Chopra G, Olson MR, Zhao B, Afzali B, Kazemian M. 2019. Integrated pan-cancer map of EBV-associated neoplasms reveals functional host-virus interactions. Cancer Res 79:6010–6023. doi:10.1158/0008-5472.CAN-19-0615 31481499 PMC6891172

[55] Wong Y, Meehan MT, Burrows SR, Doolan DL, Miles JJ. 2022. Estimating the global burden of Epstein–Barr virus-related cancers. J Cancer Res Clin Oncol 148:31–46. doi:10.1007/s00432-021-03824-y 34705104 PMC8752571

[56] Al-Anazi AE, Alanazi BS, Alshanbari HM, Masuadi E, Hamed ME, Dandachi I, Alkathiri A, Hanif A, Nour I, Fatani H, Alsaran H, AlKhareeb F, Al Zahrani A, Alsharm AA, Eifan S, Alosaimi B. 2023. Increased prevalence of EBV infection in nasopharyngeal carcinoma patients: a six-year cross-sectional study. Cancers (Basel) 15:643. doi:10.3390/cancers15030643 36765601 PMC9913071

[57] Donzel M, Bonjour M, Combes J-D, Broussais F, Sesques P, Traverse-Glehen A, de Martel C. 2022. Lymphomas associated with Epstein-Barr virus infection in 2020: results from a large, unselected case series in France. EClinicalMedicine 54:101674. doi:10.1016/j.eclinm.2022.101674 36204003 PMC9531037

[58] Farisyi MA, Sufiawati I. 2020. Detection of Epstein–Barr virus DNA in saliva of HIV-1-infected individuals with oral hairy leukoplakia. Oral Dis 26:158–160. doi:10.1111/odi.13400 32862526

[59] Soldan SS, Lieberman PM. 2023. Epstein–Barr virus and multiple sclerosis. Nat Rev Microbiol 21:51–64. doi:10.1038/s41579-022-00770-5 35931816 PMC9362539

[60] Tarbouriech N, Buisson M, Géoui T, Daenke S, Cusack S, Burmeister WP. 2006. Structural genomics of the Epstein–Barr virus. Acta Crystallogr D Biol Crystallogr 62:1276–1285. doi:10.1107/S0907444906030034 17001105

[61] Moss WN, Lee N, Pimienta G, Steitz JA. 2014. RNA families in Epstein-Barr virus. RNA Biol 11:10–17. doi:10.4161/rna.27488 24441309 PMC3929418

[62] Skalsky RL, Cullen BR. 2015. EBV noncoding RNAs. Curr Top Microbiol Immunol 391:181–217. doi:10.1007/978-3-319-22834-1_6 26428375 PMC5685189

[63] Farrell PJ. 2019. Epstein-Barr virus and cancer. Annu Rev Pathol 14:29–53. doi:10.1146/annurev-pathmechdis-012418-013023 30125149

[64] Chesnokova LS, Jiang R, Hutt-Fletcher LM. 2015. Viral entry. Curr Top Microbiol Immunol 391:221–235. doi:10.1007/978-3-319-22834-1_7 26428376

[65] Jean-Pierre V, Lupo J, Buisson M, Morand P, Germi R. 2021. Main targets of interest for the development of a prophylactic or therapeutic Epstein-Barr virus vaccine. Front Microbiol 12:701611. doi:10.3389/fmicb.2021.701611 34239514 PMC8258399

[66] Miller N, Hutt-Fletcher LM. 1992. Epstein-Barr virus enters B cells and epithelial cells by different routes. J Virol 66:3409–3414. doi:10.1128/JVI.66.6.3409-3414.1992 1316456 PMC241121

[67] Valencia SM, Hutt-Fletcher LM. 2012. Important but differential roles for actin in trafficking of Epstein-Barr virus in B cells and epithelial cells. J Virol 86:2–10. doi:10.1128/JVI.05883-11 22031939 PMC3255874

[68] Lee C-P, Chen M-R. 2021. Conquering the nuclear envelope barriers by EBV lytic replication. Viruses 13:702. doi:10.3390/v13040702 33919628 PMC8073350

[69] Murata T. 2014. Regulation of Epstein–Barr virus reactivation from latency. Microbiol Immunol 58:307–317. doi:10.1111/1348-0421.12155 24786491

[70] Hammerschmidt W. 2015. The epigenetic life cycle of Epstein-Barr virus. Curr Top Microbiol Immunol 390:103–117. doi:10.1007/978-3-319-22822-8_6 26424645

[71] Sausen DG, Bhutta MS, Gallo ES, Dahari H, Borenstein R. 2021. Stress-induced Epstein-Barr virus reactivation. Biomolecules 11:1380. doi:10.3390/biom11091380 34572593 PMC8470332

[72] Gruhne B, Sompallae R, Masucci MG. 2009. Three Epstein-Barr virus latency proteins independently promote genomic instability by inducing DNA damage, inhibiting DNA repair and inactivating cell cycle checkpoints. Oncogene 28:3997–4008. doi:10.1038/onc.2009.258 19718051

[73] Deng W, Pang PS, Tsang CM, Hau PM, Yip YL, Cheung ALM, Tsao SW. 2012. Epstein-Barr virus-encoded latent membrane protein 1 impairs G2 checkpoint in human nasopharyngeal epithelial cells through defective Chk1 activation. PLoS One 7:e39095. doi:10.1371/journal.pone.0039095 22761726 PMC3382577

[74] Dolcetti R, Dal Col J, Martorelli D, Carbone A, Klein E. 2013. Interplay among viral antigens, cellular pathways and tumor microenvironment in the pathogenesis of EBV-driven lymphomas. Semin Cancer Biol 23:441–456. doi:10.1016/j.semcancer.2013.07.005 23917255

[75] Tsao S-W, Tsang CM, To K-F, Lo K-W. 2015. The role of Epstein–Barr virus in epithelial malignancies. J Pathol 235:323–333. doi:10.1002/path.4448 25251730 PMC4280676

[76] Tsang CM, Lui VWY, Bruce JP, Pugh TJ, Lo KW. 2020. Translational genomics of nasopharyngeal cancer. Semin Cancer Biol 61:84–100. doi:10.1016/j.semcancer.2019.09.006 31521748

[77] Shumilov A, Tsai M-H, Schlosser YT, Kratz A-S, Bernhardt K, Fink S, Mizani T, Lin X, Jauch A, Mautner J, Kopp-Schneider A, Feederle R, Hoffmann I, Delecluse H-J. 2017. Epstein-Barr virus particles induce centrosome amplification and chromosomal instability. Nat Commun 8:14257. doi:10.1038/ncomms14257 28186092 PMC5309802

[78] Hurley EA, Agger S, McNeil JA, Lawrence JB, Calendar A, Lenoir G, Thorley-Lawson DA. 1991. When Epstein-Barr virus persistently infects B-cell lines, it frequently integrates. J Virol 65:1245–1254. doi:10.1128/JVI.65.3.1245-1254.1991 1847452 PMC239896

[79] Ohshima K, Suzumiya J, Kanda M, Kato A, Kikuchi M. 1998. Integrated and episomal forms of Epstein–Barr virus (EBV) in EBV associated disease. Cancer Lett 122:43–50. doi:10.1016/S0304-3835(97)00368-6 9464490

[80] Xiao K, Yu Z, Li X, Li X, Tang K, Tu C, Qi P, Liao Q, Chen P, Zeng Z, Li G, Xiong W. 2016. Genome-wide analysis of Epstein-Barr Virus (EBV) integration and strain in C666-1 and Raji cells. J Cancer 7:214–224. doi:10.7150/jca.13150 26819646 PMC4716855

[81] Peng R-J, Han B-W, Cai Q-Q, Zuo X-Y, Xia T, Chen J-R, Feng L-N, Lim JQ, Chen S-W, Zeng M-S, Guo Y-M, Li B, Xia X-J, Xia Y, Laurensia Y, Chia BKH, Huang H-Q, Young KH, Lim ST, Ong CK, Zeng Y-X, Bei J-X. 2019. Genomic and transcriptomic landscapes of Epstein-Barr virus in extranodal natural killer T-cell lymphoma. Leukemia 33:1451–1462. doi:10.1038/s41375-018-0324-5 30546078 PMC6756073

[82] Koliais SI. 1979. Mode of integration of Epstein-Barr virus genome into host DNA in Burkitt lymphoma cells. J Gen Virol 44:573–576. doi:10.1099/0022-1317-44-2-573 230297

[83] Anvret M, Karlsson A, Bjursell G. 1984. Evidence for integrated EBV genomes in Raji cellular DNA. Nucleic Acids Res 12:1149–1161. doi:10.1093/nar/12.2.1149 6320116 PMC318562

[84] Kieff E, Hennessy K, Fennewald S, Matsuo T, Dambaugh T, Heller M, Hummel M. 1985. Biochemistry of latent Epstein-Barr virus infection and associated cell growth transformation, p 323–339. IARC scientific publications. 2998995

[85] Xu M, Zhang W-L, Zhu Q, Zhang S, Yao Y-Y, Xiang T, Feng Q-S, Zhang Z, Peng R-J, Jia W-H, He G-P, Feng L, Zeng Z-L, Luo B, Xu R-H, Zeng M-S, Zhao W-L, Chen S-J, Zeng Y-X, Jiao Y. 2019. Genome-wide profiling of Epstein-Barr virus integration by targeted sequencing in Epstein-Barr virus associated malignancies. Theranostics 9:1115–1124. doi:10.7150/thno.29622 30867819 PMC6401403

[86] Delecluse HJ, Bartnizke S, Hammerschmidt W, Bullerdiek J, Bornkamm GW. 1993. Episomal and integrated copies of Epstein-Barr virus coexist in Burkitt lymphoma cell lines. J Virol 67:1292–1299. doi:10.1128/JVI.67.3.1292-1299.1993 8382295 PMC237496

[87] Kripalani-Joshi S, Law HY. 1994. Identification of integrated Epstein-Barr virus in nasopharyngeal carcinoma using pulse field gel electrophoresis. Int J Cancer 56:187–192. doi:10.1002/ijc.2910560207 8314299

[88] Takakuwa T, Luo W-J, Ham MF, Sakane-Ishikawa F, Wada N, Aozasa K. 2004. Integration of Epstein-Barr virus into chromosome 6q15 of Burkitt lymphoma cell line (Raji) induces loss of BACH2 expression. Am J Pathol 164:967–974. doi:10.1016/S0002-9440(10)63184-7 14982850 PMC1614712

[89] Zhang L, Wang R, Xie Z. 2022. The roles of DNA methylation on the promotor of the Epstein–Barr virus (EBV) gene and the genome in patients with EBV-associated diseases. Appl Microbiol Biotechnol 106:4413–4426. doi:10.1007/s00253-022-12029-3 35763069 PMC9259528

[90] Luo W-J, Takakuwa T, Ham MF, Wada N, Liu A, Fujita S, Sakane-Ishikawa E, Aozasa K. 2004. Epstein-Barr virus is integrated between REL and BCL-11A in American Burkitt lymphoma cell line (NAB-2). Lab Invest 84:1193–1199. doi:10.1038/labinvest.3700152 15241441

[91] Janjetovic S, Hinke J, Balachandran S, Akyüz N, Behrmann P, Bokemeyer C, Dierlamm J, Murga Penas EM. 2022. Non-random pattern of integration for epstein-barr virus with preference for gene-poor genomic chromosomal regions into the genome of burkitt lymphoma cell lines. Viruses 14:86. doi:10.3390/v14010086 35062290 PMC8781420

[92] Tang D, Li B, Xu T, Hu R, Tan D, Song X, Jia P, Zhao Z. 2020. VISDB: a manually curated database of viral integration sites in the human genome. Nucleic Acids Res 48:D633–D641. doi:10.1093/nar/gkz867 31598702 PMC6943068

[93] Gao J, Luo X, Tang K, Li X, Li G. 2006. Epstein-Barr virus integrates frequently into chromosome 4q, 2q, 1q and 7q of Burkitt’s lymphoma cell line (Raji). J Virol Methods 136:193–199. doi:10.1016/j.jviromet.2006.05.013 16806502

[94] Gasenko E, Isajevs S, Camargo MC, Offerhaus GJA, Polaka I, Gulley ML, Skapars R, Sivins A, Kojalo I, Kirsners A, Santare D, Pavlova J, Sjomina O, Liepina E, Tzivian L, Rabkin CS, Leja M. 2019. Clinicopathological characteristics of Epstein-Barr virus-positive gastric cancer in Latvia. Eur J Gastroenterol Hepatol 31:1328–1333. doi:10.1097/MEG.0000000000001521 31569122 PMC8560222

[95] He C-Y, Qiu M-Z, Yang X-H, Zhou D-L, Ma J-J, Long Y-K, Ye Z-L, Xu B-H, Zhao Q, Jin Y, Lu S-X, Wang Z-Q, Guan W-L, Zhao B-W, Zhou Z-W, Shao J-Y, Xu R-H. 2020. Classification of gastric cancer by EBV status combined with molecular profiling predicts patient prognosis. Clin Transl Med 10:353–362. doi:10.1002/ctm2.32 32508039 PMC7240851

[96] Qiu M-Z, He C-Y, Lu S-X, Guan W-L, Wang F, Wang X-J, Jin Y, Wang F-H, Li Y-H, Shao J-Y, Zhou Z-W, Yun J-P, Xu R-H. 2020. Prospective observation: clinical utility of plasma Epstein-Barr virus DNA load in EBV-associated gastric carcinoma patients. Int J Cancer 146:272–280. doi:10.1002/ijc.32490 31162842

[97] PaVE. 2025. Available from: https://pave.niaid.nih.gov. Retrieved 06 Mar 2025.

[98] Burchell AN, Winer RL, de Sanjosé S, Franco EL. 2006. Chapter 6: epidemiology and transmission dynamics of genital HPV infection. Vaccine (Auckl) 24:S3 doi:10.1016/j.vaccine.2006.05.031 16950018

[99] Bruni L, Diaz M, Castellsagué X, Ferrer E, Bosch FX, de Sanjosé S. 2010. Cervical human papillomavirus prevalence in 5 continents: meta-analysis of 1 million women with normal cytological findings. J Infect Dis 202:1789–1799. doi:10.1086/657321 21067372

[100] Human papillomavirus and cancer. 2024. Available from: https://www.who.int/news-room/fact-sheets/detail/human-papilloma-virus-and-cancer. Retrieved Jul 2025.

[101] Bruni L, Albero G, Rowley J, Alemany L, Arbyn M, Giuliano AR, Markowitz LE, Broutet N, Taylor M. 2023. Global and regional estimates of genital human papillomavirus prevalence among men: a systematic review and meta-analysis. Lancet Glob Health 11:e1345–e1362. doi:10.1016/S2214-109X(23)00305-4 37591583 PMC10447222

[102] Malik H, Khan FH, Ahsan H. 2014. Human papillomavirus: current status and issues of vaccination. Arch Virol 159:199–205. doi:10.1007/s00705-013-1827-z 24022639

[103] Serrano B, Albero G, Bruni L 2025. Fact Sheet World 262. Available from: https://www.hpvworld.com/articles/fact-sheet-world. Retrieved 6 Jun 2025.

[104] de Martel C, Plummer M, Vignat J, Franceschi S. 2017. Worldwide burden of cancer attributable to HPV by site, country and HPV type. Int J Cancer 141:664–670. doi:10.1002/ijc.30716 28369882 PMC5520228

[105] Ndon S, Singh A, Ha PK, Aswani J, Chan J-K, Xu MJ. 2023. Human papillomavirus-associated oropharyngeal cancer: global epidemiology and public policy implications. Cancers (Basel) 15:4080. doi:10.3390/cancers15164080 37627108 PMC10452639

[106] de Sanjosé S, Brotons M, Pavón MA. 2018. The natural history of human papillomavirus infection. Best Pract Res Clin Obstet Gynaecol 47:2–13. doi:10.1016/j.bpobgyn.2017.08.015 28964706

[107] Mikuličić S, Strunk J, Florin L. 2021. HPV16 entry into epithelial cells: running a gauntlet. Viruses 13:2460. doi:10.3390/v13122460 34960729 PMC8706107

[108] McBride AA. 2008. Replication and partitioning of papillomavirus genomes. Adv Virus Res 72:155–205. doi:10.1016/S0065-3527(08)00404-1 19081491 PMC3151303

[109] Radley D, Saah A, Stanley M. 2016. Persistent infection with human papillomavirus 16 or 18 is strongly linked with high-grade cervical disease. Hum Vaccin Immunother 12:768–772. doi:10.1080/21645515.2015.1088616 26383553 PMC4964639

[110] Moscicki A-B, Shiboski S, Hills NK, Powell KJ, Jay N, Hanson EN, Miller S, Canjura-Clayton KL, Farhat S, Broering JM, Darragh TM. 2004. Regression of low-grade squamous intra-epithelial lesions in young women. Lancet 364:1678–1683. doi:10.1016/S0140-6736(04)17354-6 15530628

[111] Ostör AG. 1993. Natural history of cervical intraepithelial neoplasia: a critical review. Int J Gynecol Pathol 12:186–192. 8463044

[112] Basu P, Taghavi K, Hu S-Y, Mogri S, Joshi S. 2018. Management of cervical premalignant lesions. Curr Probl Cancer 42:129–136. doi:10.1016/j.currproblcancer.2018.01.010 29428790

[113] Bhattacharjee R, Das SS, Biswal SS, Nath A, Das D, Basu A, Malik S, Kumar L, Kar S, Singh SK, Upadhye VJ, Iqbal D, Almojam S, Roychoudhury S, Ojha S, Ruokolainen J, Jha NK, Kesari KK. 2022. Mechanistic role of HPV-associated early proteins in cervical cancer: molecular pathways and targeted therapeutic strategies. Crit Rev Oncol Hematol 174:103675. doi:10.1016/j.critrevonc.2022.103675 35381343

[114] Scheffner M, Huibregtse JM, Vierstra RD, Howley PM. 1993. The HPV-16 E6 and E6-AP complex functions as a ubiquitin-protein ligase in the ubiquitination of p53. Cell 75:495–505. doi:10.1016/0092-8674(93)90384-3 8221889

[115] Beaudenon S, Huibregtse JM. 2008. HPV E6, E6AP and cervical cancer. BMC Biochem 9:S4. doi:10.1186/1471-2091-9-S1-S4 19007434 PMC2582798

[116] Mortensen F, Schneider D, Barbic T, Sladewska-Marquardt A, Kühnle S, Marx A, Scheffner M. 2015. Role of ubiquitin and the HPV E6 oncoprotein in E6AP-mediated ubiquitination. Proc Natl Acad Sci USA 112:9872–9877. doi:10.1073/pnas.1505923112 26216987 PMC4538620

[117] Zimmermann H, Degenkolbe R, Bernard HU, O’Connor MJ. 1999. The human papillomavirus type 16 E6 oncoprotein can down-regulate p53 activity by targeting the transcriptional coactivator CBP/p300. J Virol 73:6209–6219. doi:10.1128/JVI.73.8.6209-6219.1999 10400710 PMC112697

[118] Patel D, Huang SM, Baglia LA, McCance DJ. 1999. The E6 protein of human papillomavirus type 16 binds to and inhibits co-activation by CBP and p300. EMBO J 18:5061–5072. doi:10.1093/emboj/18.18.5061 10487758 PMC1171577

[119] Yoshimatsu Y, Nakahara T, Tanaka K, Inagawa Y, Narisawa-Saito M, Yugawa T, Ohno S-I, Fujita M, Nakagama H, Kiyono T. 2017. Roles of the PDZ-binding motif of HPV 16 E6 protein in oncogenic transformation of human cervical keratinocytes. Cancer Sci 108:1303–1309. doi:10.1111/cas.13264 28440909 PMC5497797

[120] Ronco LV, Karpova AY, Vidal M, Howley PM. 1998. Human papillomavirus 16 E6 oncoprotein binds to interferon regulatory factor-3 and inhibits its transcriptional activity. Genes Dev 12:2061–2072. doi:10.1101/gad.12.13.2061 9649509 PMC316980

[121] Liu X, Dakic A, Zhang Y, Dai Y, Chen R, Schlegel R. 2009. HPV E6 protein interacts physically and functionally with the cellular telomerase complex. Proc Natl Acad Sci USA 106:18780–18785. doi:10.1073/pnas.0906357106 19843693 PMC2773972

[122] Münger K, Howley PM. 2002. Human papillomavirus immortalization and transformation functions. Virus Res 89:213–228. doi:10.1016/s0168-1702(02)00190-9 12445661

[123] Longworth MS, Laimins LA. 2004. The binding of histone deacetylases and the integrity of zinc finger-like motifs of the E7 protein are essential for the life cycle of human papillomavirus type 31. J Virol 78:3533–3541. doi:10.1128/JVI.78.7.3533-3541.2004 15016876 PMC371089

[124] McLaughlin-Drubin ME, Münger K. 2009. The human papillomavirus E7 oncoprotein. Virology (Auckl) 384:335–344. doi:10.1016/j.virol.2008.10.006 PMC2661820 19007963

[125] Park JS, Kim EJ, Kwon HJ, Hwang ES, Namkoong SE, Um SJ. 2000. Inactivation of interferon regulatory factor-1 tumor suppressor protein by HPV E7 oncoprotein. Implication for the E7-mediated immune evasion mechanism in cervical carcinogenesis. J Biol Chem 275:6764–6769. doi:10.1074/jbc.275.10.6764 10702232

[126] Um S-J, Rhyu J-W, Kim E-J, Jeon K-C, Hwang E-S, Park J-S. 2002. Abrogation of IRF-1 response by high-risk HPV E7 protein in vivo. Cancer Lett 179:205–212. doi:10.1016/s0304-3835(01)00871-0 11888675

[127] Tomakidi P, Cheng H, Kohl A, Komposch G, Alonso A. 2000. Modulation of the epidermal growth factor receptor by the human papillomavirus type 16 E5 protein in raft cultures of human keratinocytes. Eur J Cell Biol 79:407–412. doi:10.1078/0171-9335-00060 10928456

[128] Wasson CW, Morgan EL, Müller M, Ross RL, Hartley M, Roberts S, Macdonald A. 2017. Human papillomavirus type 18 E5 oncogene supports cell cycle progression and impairs epithelial differentiation by modulating growth factor receptor signalling during the virus life cycle. Oncotarget 8:103581–103600. doi:10.18632/oncotarget.21658 29262586 PMC5732752

[129] Oh J-M, Kim S-H, Cho E-A, Song Y-S, Kim W-H, Juhnn Y-S. 2010. Human papillomavirus type 16 E5 protein inhibits hydrogen peroxide-induced apoptosis by stimulating ubiquitin–proteasome-mediated degradation of Bax in human cervical cancer cells. Carcinogenesis 31:402–410. doi:10.1093/carcin/bgp318 20015862

[130] Kim S-H, Juhnn Y-S, Kang S, Park S-W, Sung M-W, Bang Y-J, Song Y-S. 2006. Human papillomavirus 16 E5 up-regulates the expression of vascular endothelial growth factor through the activation of epidermal growth factor receptor, MEK/ ERK1,2 and PI3K/Akt. Cell Mol Life Sci 63:930–938. doi:10.1007/s00018-005-5561-x 16596339 PMC11136283

[131] Ilahi NE, Bhatti A. 2020. Impact of HPV E5 on viral life cycle via EGFR signaling. Microb Pathog 139:103923. doi:10.1016/j.micpath.2019.103923 31836496

[132] Kim S-H, Oh J-M, No J-H, Bang Y-J, Juhnn Y-S, Song Y-S. 2009. Involvement of NF-κB and AP-1 in COX-2 upregulation by human papillomavirus 16 E5 oncoprotein. Carcinogenesis 30:753–757. doi:10.1093/carcin/bgp066 19321801

[133] Hatano T, Sano D, Takahashi H, Oridate N. 2021. Pathogenic role of immune evasion and integration of human papillomavirus in oropharyngeal cancer. Microorganisms 9:891. doi:10.3390/microorganisms9050891 33919460 PMC8143538

[134] Moody CA, Laimins LA. 2009. Human papillomaviruses activate the ATM DNA damage pathway for viral genome amplification upon differentiation. PLoS Pathog 5:e1000605. doi:10.1371/journal.ppat.1000605 19798429 PMC2745661

[135] Hong S, Laimins LA. 2013. Regulation of the life cycle of HPVs by differentiation and the DNA damage response. Future Microbiol 8:1547–1557. doi:10.2217/fmb.13.127 24266355 PMC3951404

[136] Spriggs CC, Laimins LA. 2017. FANCD2 binds human papillomavirus genomes and associates with a distinct set of DNA repair proteins to regulate viral replication. mBio 8:e02340-16. doi:10.1128/mBio.02340-16 28196964 PMC5312087

[137] Kono T, Laimins L. 2021. Genomic instability and DNA damage repair pathways induced by human papillomaviruses. Viruses 13:1821. doi:10.3390/v13091821 34578402 PMC8472259

[138] Williams VM, Filippova M, Filippov V, Payne KJ, Duerksen-Hughes P. 2014. Human papillomavirus type 16 E6* induces oxidative stress and DNA damage. J Virol 88:6751–6761. doi:10.1128/JVI.03355-13 24696478 PMC4054338

[139] Duensing S, Lee LY, Duensing A, Basile J, Piboonniyom S, Gonzalez S, Crum CP, Munger K. 2000. The human papillomavirus type 16 E6 and E7 oncoproteins cooperate to induce mitotic defects and genomic instability by uncoupling centrosome duplication from the cell division cycle. Proc Natl Acad Sci USA 97:10002–10007. doi:10.1073/pnas.170093297 10944189 PMC27652

[140] Duensing S, Duensing A, Crum CP, Münger K. 2001. Human papillomavirus type 16 E7 oncoprotein-induced abnormal centrosome synthesis is an early event in the evolving malignant phenotype. Cancer Res 61:2356–2360. 11289095

[141] Korzeniewski N, Spardy N, Duensing A, Duensing S. 2011. Genomic instability and cancer: lessons learned from human papillomaviruses. Cancer Lett 305:113–122. doi:10.1016/j.canlet.2010.10.013 21075512 PMC3046211

[142] Hudelist G, Manavi M, Pischinger KID, Watkins-Riedel T, Singer CF, Kubista E, Czerwenka KF. 2004. Physical state and expression of HPV DNA in benign and dysplastic cervical tissue: different levels of viral integration are correlated with lesion grade. Gynecol Oncol 92:873–880. doi:10.1016/j.ygyno.2003.11.035 14984955

[143] Briolat J, Dalstein V, Saunier M, Joseph K, Caudroy S, Prétet J-L, Birembaut P, Clavel C. 2007. HPV prevalence, viral load and physical state of HPV-16 in cervical smears of patients with different grades of CIN. Int J Cancer 121:2198–2204. doi:10.1002/ijc.22959 17657742

[144] Khoury JD, Tannir NM, Williams MD, Chen Y, Yao H, Zhang J, Thompson EJ, TCGA Network, Meric-Bernstam F, Medeiros LJ, Weinstein JN, Su X. 2013. Landscape of DNA virus associations across human malignant cancers: analysis of 3,775 cases using RNA-Seq. J Virol 87:8916–8926. doi:10.1128/JVI.00340-13 23740984 PMC3754044

[145] Vojtechova Z, Sabol I, Salakova M, Turek L, Grega M, Smahelova J, Vencalek O, Lukesova E, Klozar J, Tachezy R. 2016. Analysis of the integration of human papillomaviruses in head and neck tumours in relation to patients’ prognosis. Int J Cancer 138:386–395. doi:10.1002/ijc.29712 26239888

[146] Cancer Genome Atlas Research Network, Albert Einstein College of Medicine, Analytical Biological Services, Barretos Cancer Hospital, Baylor College of Medicine, Beckman Research Institute of City of Hope, Buck Institute for Research on Aging Canada’s Michael Smith Genome Sciences Centre Harvard Medical School, Helen F. Graham Cancer Center & Research Institute at Christiana Care Health Services, et al.. 2017. Integrated genomic and molecular characterization of cervical cancer. Nature 543:378–384. doi:10.1038/nature21386 28112728 PMC5354998

[147] Tang KD, Baeten K, Kenny L, Frazer IH, Scheper G, Punyadeera C. 2019. Unlocking the potential of saliva-based test to detect HPV-16-driven oropharyngeal cancer. Cancers (Basel) 11:473. doi:10.3390/cancers11040473 30987261 PMC6521163

[148] Mainguené J, Vacher S, Kamal M, Hamza A, Masliah-Planchon J, Baulande S, Ibadioune S, Borcoman E, Cacheux W, Calugaru V, et al.. 2022. Human papilloma virus integration sites and genomic signatures in head and neck squamous cell carcinoma. Mol Oncol 16:3001–3016. doi:10.1002/1878-0261.13219 35398964 PMC9394244

[149] Gray E, Pett MR, Ward D, Winder DM, Stanley MA, Roberts I, Scarpini CG, Coleman N. 2010. In vitro progression of human papillomavirus 16 episome-associated cervical neoplasia displays fundamental similarities to integrant-associated carcinogenesis. Cancer Res 70:4081–4091. doi:10.1158/0008-5472.CAN-09-3335 20442284 PMC2872760

[150] Rossi NM, Dai J, Xie Y, Wangsa D, Heselmeyer-Haddad K, Lou H, Boland JF, Yeager M, Orozco R, Freites EA, Mirabello L, Gharzouzi E, Dean M. 2023. Extrachromosomal amplification of human papillomavirus episomes is a mechanism of cervical carcinogenesis. Cancer Res 83:1768–1781. doi:10.1158/0008-5472.CAN-22-3030 36971511 PMC10239328

[151] Chaiwongkot A, Vinokurova S, Pientong C, Ekalaksananan T, Kongyingyoes B, Kleebkaow P, Chumworathayi B, Patarapadungkit N, Reuschenbach M, von Knebel Doeberitz M. 2013. Differential methylation of E2 binding sites in episomal and integrated HPV 16 genomes in preinvasive and invasive cervical lesions. Int J Cancer 132:2087–2094. doi:10.1002/ijc.27906 23065631

[152] Pokrývková B, Saláková M, Šmahelová J, Vojtěchová Z, Novosadová V, Tachezy R. 2019. Detailed characteristics of tonsillar tumors with extrachromosomal or integrated form of human papillomavirus. Viruses 12:42. doi:10.3390/v12010042 31905862 PMC7019694

[153] Cheung JLK, Cheung T-H, Yu MY, Chan PKS. 2013. Virological characteristics of cervical cancers carrying pure episomal form of HPV16 genome. Gynecol Oncol 131:374–379. doi:10.1016/j.ygyno.2013.08.026 24012799

[154] Ren S, Gaykalova DA, Guo T, Favorov AV, Fertig EJ, Tamayo P, Callejas-Valera JL, Allevato M, Gilardi M, Santos J, Fukusumi T, Sakai A, Ando M, Sadat S, Liu C, Xu G, Fisch KM, Wang Z, Molinolo AA, Gutkind JS, Ideker T, Koch WM, Califano JA. 2020. HPV E2, E4, E5 drive alternative carcinogenic pathways in HPV positive cancers. Oncogene 39:6327–6339. doi:10.1038/s41388-020-01431-8 32848210 PMC7529583

[155] Williams VM, Filippova M, Soto U, Duerksen-Hughes PJ. 2011. HPV-DNA integration and carcinogenesis: putative roles for inflammation and oxidative stress. Future Virol 6:45–57. doi:10.2217/fvl.10.73 21318095 PMC3037184

[156] Visalli G, Riso R, Facciolà A, Mondello P, Caruso C, Picerno I, Di Pietro A, Spataro P, Bertuccio MP. 2016. Higher levels of oxidative DNA damage in cervical cells are correlated with the grade of dysplasia and HPV infection. J Med Virol 88:336–344. doi:10.1002/jmv.24327 26174792

[157] Kondo S, Wakae K, Wakisaka N, Nakanishi Y, Ishikawa K, Komori T, Moriyama-Kita M, Endo K, Murono S, Wang Z, Kitamura K, Nishiyama T, Yamaguchi K, Shigenobu S, Muramatsu M, Yoshizaki T. 2017. APOBEC3A associates with human papillomavirus genome integration in oropharyngeal cancers. Oncogene 36:1687–1697. doi:10.1038/onc.2016.335 27694899

[158] Zapatka M, Borozan I, Brewer DS, Iskar M, Grundhoff A, Alawi M, Desai N, Sültmann H, Moch H, Cooper CS, Eils R, Ferretti V, Lichter P, PCAWG Pathogens, PCAWG Consortium. 2020. The landscape of viral associations in human cancers. Nat Genet 52:320–330. doi:10.1038/s41588-019-0558-9 32025001 PMC8076016

[159] Hu Z, Zhu D, Wang W, Li W, Jia W, Zeng X, Ding W, Yu L, Wang X, Wang L, et al.. 2015. Genome-wide profiling of HPV integration in cervical cancer identifies clustered genomic hot spots and a potential microhomology-mediated integration mechanism. Nat Genet 47:158–163. doi:10.1038/ng.3178 25581428

[160] Zhang R, Shen C, Zhao L, Wang J, McCrae M, Chen X, Lu F. 2016. Dysregulation of host cellular genes targeted by human papillomavirus (HPV) integration contributes to HPV-related cervical carcinogenesis. Int J Cancer 138:1163–1174. doi:10.1002/ijc.29872 26417997 PMC5057319

[161] Kamal M, Lameiras S, Deloger M, Morel A, Vacher S, Lecerf C, Dupain C, Jeannot E, Girard E, Baulande S, Dubot C, Kenter G, Jordanova ES, Berns EMJJ, Bataillon G, Popovic M, Rouzier R, Cacheux W, Le Tourneau C, Nicolas A, Servant N, Scholl SM, Bièche I, RAIDs Consortium. 2021. Human papilloma virus (HPV) integration signature in Cervical cancer: identification of MACROD2 gene as HPV hot spot integration site. Br J Cancer 124:777–785. doi:10.1038/s41416-020-01153-4 33191407 PMC7884736

[162] Fan J, Fu Y, Peng W, Li X, Shen Y, Guo E, Lu F, Zhou S, Liu S, Yang B, et al.. 2023. Multi-omics characterization of silent and productive HPV integration in cervical cancer. Cell Genom 3:100211. doi:10.1016/j.xgen.2022.100211 36777180 PMC9903858

[163] Zeng X, Wang Y, Liu B, Rao X, Cao C, Peng F, Zhi W, Wu P, Peng T, Wei Y, Chu T, Xu M, Xu Y, Ding W, Li G, Lin S, Wu P. 2023. Multi-omics data reveals novel impacts of human papillomavirus integration on the epigenomic and transcriptomic signatures of cervical tumorigenesis. J Med Virol 95:e28789. doi:10.1002/jmv.28789 37212325

[164] Thorland EC, Myers SL, Persing DH, Sarkar G, McGovern RM, Gostout BS, Smith DI. 2000. Human papillomavirus type 16 integrations in cervical tumors frequently occur in common fragile sites. Cancer Res 60:5916–5921. 11085503

[165] Bodelon C, Untereiner ME, Machiela MJ, Vinokurova S, Wentzensen N. 2016. Genomic characterization of viral integration sites in HPV-related cancers. Int J Cancer 139:2001–2011. doi:10.1002/ijc.30243 27343048 PMC6749823

[166] Gao G, Johnson SH, Vasmatzis G, Pauley CE, Tombers NM, Kasperbauer JL, Smith DI. 2017. Common fragile sites (CFS) and extremely large CFS genes are targets for human papillomavirus integrations and chromosome rearrangements in oropharyngeal squamous cell carcinoma. Genes Chromosomes Cancer 56:59–74. doi:10.1002/gcc.22415 27636103

[167] Wentzensen N, Vinokurova S, von Knebel Doeberitz M. 2004. Systematic review of genomic integration sites of human papillomavirus genomes in epithelial dysplasia and invasive cancer of the female lower genital tract. Cancer Res 64:3878–3884. doi:10.1158/0008-5472.CAN-04-0009 15172997

[168] Ragin CCR, Reshmi SC, Gollin SM. 2004. Mapping and analysis of HPV16 integration sites in a head and neck cancer cell line. Intl J Cancer 110:701–709. doi:10.1002/ijc.20193 15146560

[169] Akagi K, Li J, Broutian TR, Padilla-Nash H, Xiao W, Jiang B, Rocco JW, Teknos TN, Kumar B, Wangsa D, He D, Ried T, Symer DE, Gillison ML. 2014. Genome-wide analysis of HPV integration in human cancers reveals recurrent, focal genomic instability. Genome Res 24:185–199. doi:10.1101/gr.164806.113 24201445 PMC3912410

[170] Zhao J, Zheng W, Wang L, Jiang H, Wang X, Hou J, Xu A, Cong J. 2023. Human papillomavirus (HPV) integration signature in cervical lesions: identification of MACROD2 gene as HPV hot spot integration site. Arch Gynecol Obstet 307:1115–1123. doi:10.1007/s00404-022-06748-1 36008642

[171] Parfenov M, Pedamallu CS, Gehlenborg N, Freeman SS, Danilova L, Bristow CA, Lee S, Hadjipanayis AG, Ivanova EV, Wilkerson MD, et al.. 2014. Characterization of HPV and host genome interactions in primary head and neck cancers. Proc Natl Acad Sci USA 111:15544–15549. doi:10.1073/pnas.1416074111 25313082 PMC4217452

[172] Olthof NC, Speel E-J, Kolligs J, Haesevoets A, Henfling M, Ramaekers FCS, Preuss SF, Drebber U, Wieland U, Silling S, Lam WL, Vucic EA, Kremer B, Klussmann J-P, Huebbers CU. 2014. Comprehensive analysis of HPV16 integration in OSCC reveals no significant impact of physical status on viral oncogene and virally disrupted human gene expression. PLoS One 9:e88718. doi:10.1371/journal.pone.0088718 24586376 PMC3933331

[173] Tian R, Huang Z, Li L, Yuan J, Zhang Q, Meng L, Lang B, Hong Y, Zhong C, Tian X, Cui Z, Jin Z, Liu J, Huang Z, Wang Y, Chen Y, Hu Z. 2023. HPV integration generates a cellular super-enhancer which functions as ecDNA to regulate genome-wide transcription. Nucleic Acids Res 51:4237–4251. doi:10.1093/nar/gkad105 36864748 PMC10201430

[174] Hatano T, Sano D, Takahashi H, Hyakusoku H, Isono Y, Shimada S, Sawakuma K, Takada K, Oikawa R, Watanabe Y, Yamamoto H, Itoh F, Myers JN, Oridate N. 2017. Identification of human papillomavirus (HPV) 16 DNA integration and the ensuing patterns of methylation in HPV-associated head and neck squamous cell carcinoma cell lines. Int J Cancer 140:1571–1580. doi:10.1002/ijc.30589 28006857 PMC5877459

[175] Badal V, Chuang LSH, Tan E-H, Badal S, Villa LL, Wheeler CM, Li BFL, Bernard H-U. 2003. CpG methylation of human papillomavirus type 16 DNA in cervical cancer cell lines and in clinical specimens: genomic hypomethylation correlates with carcinogenic progression. J Virol 77:6227–6234. doi:10.1128/jvi.77.11.6227-6234.2003 12743279 PMC154984

[176] Rosendo-Chalma P, Antonio-Véjar V, Ortiz Tejedor JG, Ortiz Segarra J, Vega Crespo B, Bigoni-Ordóñez GD. 2024. The hallmarks of cervical cancer: molecular mechanisms induced by human papillomavirus. Biology (Basel) 13:77. doi:10.3390/biology13020077 38392296 PMC10886769

[177] Balaji H, Demers I, Wuerdemann N, Schrijnder J, Kremer B, Klussmann JP, Huebbers CU, Speel E-JM. 2021. Causes and consequences of HPV integration in head and neck squamous cell carcinomas: state of the art. Cancers (Basel) 13:4089. doi:10.3390/cancers13164089 34439243 PMC8394665

[178] Veitía D, Liuzzi J, Ávila M, Rodriguez I, Toro F, Correnti M. 2020. Association of viral load and physical status of HPV-16 with survival of patients with head and neck cancer. Ecancermedicalscience 14:1082. doi:10.3332/ecancer.2020.1082 32863876 PMC7434508

[179] Nambaru L, Meenakumari B, Swaminathan R, Rajkumar T. 2009. Prognostic significance of HPV physical status and integration sites in cervical cancer. Asian Pac J Cancer Prev 10:355–360. 19640172

[180] Koneva LA, Zhang Y, Virani S, Hall PB, McHugh JB, Chepeha DB, Wolf GT, Carey TE, Rozek LS, Sartor MA. 2018. HPV integration in HNSCC correlates with survival outcomes, immune response signatures, and candidate drivers. Mol Cancer Res 16:90–102. doi:10.1158/1541-7786.MCR-17-0153 28928286 PMC5752568

[181] Nulton TJ, Kim N-K, DiNardo LJ, Morgan IM, Windle B. 2018. Patients with integrated HPV16 in head and neck cancer show poor survival. Oral Oncol 80:52–55. doi:10.1016/j.oraloncology.2018.03.015 29706188 PMC5930384

[182] Pinatti LM, Sinha HN, Brummel CV, Goudsmit CM, Geddes TJ, Wilson GD, Akervall JA, Brenner CJ, Walline HM, Carey TE. 2021. Association of human papillomavirus integration with better patient outcomes in oropharyngeal squamous cell carcinoma. Head Neck 43:544–557. doi:10.1002/hed.26501 33073473 PMC7898416

[183] Feng H, Shuda M, Chang Y, Moore PS. 2008. Clonal integration of a polyomavirus in human Merkel cell carcinoma. Science 319:1096–1100. doi:10.1126/science.1152586 18202256 PMC2740911

[184] Wijaya WA, Liu Y, Qing Y, Li Z. 2022. Prevalence of Merkel cell polyomavirus in normal and lesional skin: a systematic review and meta-analysis. Front Oncol 12:868781. doi:10.3389/fonc.2022.868781 35392226 PMC8980839

[185] Sunshine JC, Jahchan NS, Sage J, Choi J. 2018. Are there multiple cells of origin of Merkel cell carcinoma? Oncogene 37:1409–1416. doi:10.1038/s41388-017-0073-3 29321666 PMC5854515

[186] Juan HY, Khachemoune A. 2023. A review of Merkel cell carcinoma. JAAPA 36:11–16. doi:10.1097/01.JAA.0000979460.69305.b7 37820270

[187] Jacobs D, Huang H, Olino K, Weiss S, Kluger H, Judson BL, Zhang Y. 2021. Assessment of age, period, and birth cohort effects and trends in Merkel cell carcinoma incidence in the United States. JAMA Dermatol 157:59. doi:10.1001/jamadermatol.2020.4102 33146688 PMC7643047

[188] Stang A, Becker JC, Nghiem P, Ferlay J. 2018. The association between geographic location and incidence of Merkel cell carcinoma in comparison to melanoma: an international assessment. Eur J Cancer 94:47–60. doi:10.1016/j.ejca.2018.02.003 29533867 PMC6019703

[189] Wieland U, Mauch C, Kreuter A, Krieg T, Pfister H. 2009. Merkel cell polyomavirus DNA in persons without Merkel cell carcinoma. Emerg Infect Dis 15:1496–1498. doi:10.3201/eid1509.081575 19788824 PMC2819892

[190] Foulongne V, Dereure O, Kluger N, Molès JP, Guillot B, Segondy M. 2010. Merkel cell polyomavirus DNA detection in lesional and nonlesional skin from patients with Merkel cell carcinoma or other skin diseases. Br J Dermatol 162:59–63. doi:10.1111/j.1365-2133.2009.09381.x 19678822

[191] Schowalter RM, Pastrana DV, Pumphrey KA, Moyer AL, Buck CB. 2010. Merkel cell polyomavirus and two previously unknown polyomaviruses are chronically shed from human skin. Cell Host Microbe 7:509–515. doi:10.1016/j.chom.2010.05.006 20542254 PMC2919322

[192] Bopp L, Wieland U, Hellmich M, Kreuter A, Pfister H, Silling S. 2021. Natural history of cutaneous human polyomavirus infection in healthy individuals. Front Microbiol 12:740947. doi:10.3389/fmicb.2021.740947 34733257 PMC8558461

[193] Saláková M, Košlabová E, Vojtěchová Z, Tachezy R, Šroller V. 2016. Detection of human polyomaviruses MCPyV, HPyV6, and HPyV7 in malignant and non-malignant tonsillar tissues. J Med Virol 88:695–702. doi:10.1002/jmv.24385 26381295

[194] Nicol JTJ, Robinot R, Carpentier A, Carandina G, Mazzoni E, Tognon M, Touzé A, Coursaget P. 2013. Age-specific seroprevalences of merkel cell polyomavirus, human polyomaviruses 6, 7, and 9, and trichodysplasia spinulosa-associated polyomavirus. Clin Vaccine Immunol 20:363–368. doi:10.1128/CVI.00438-12 23302741 PMC3592346

[195] Zhang C, Liu F, He Z, Deng Q, Pan Y, Liu Y, Zhang C, Ning T, Guo C, Liang Y, Xu R, Zhang L, Cai H, Ke Y. 2014. Seroprevalence of Merkel cell polyomavirus in the general rural population of Anyang, China. PLoS One 9:e106430. doi:10.1371/journal.pone.0106430 25184447 PMC4153645

[196] Šroller V, Hamšíková E, Ludvíková V, Vochozková P, Kojzarová M, Fraiberk M, Saláková M, Morávková A, Forstová J, Němečková Š. 2014. Seroprevalence rates of BKV, JCV, and MCPyV polyomaviruses in the general Czech Republic population. J Med Virol 86:1560–1568. doi:10.1002/jmv.23841 24214630

[197] Carter JJ, Daugherty MD, Qi X, Bheda-Malge A, Wipf GC, Robinson K, Roman A, Malik HS, Galloway DA. 2013. Identification of an overprinting gene in Merkel cell polyomavirus provides evolutionary insight into the birth of viral genes. Proc Natl Acad Sci USA 110:12744–12749. doi:10.1073/pnas.1303526110 23847207 PMC3732942

[198] Seo GJ, Chen CJ, Sullivan CS. 2009. Merkel cell polyomavirus encodes a microRNA with the ability to autoregulate viral gene expression. Virology (Auckl) 383:183–187. doi:10.1016/j.virol.2008.11.001 19046593

[199] Schowalter RM, Buck CB. 2013. The Merkel cell polyomavirus minor capsid protein. PLoS Pathog 9:e1003558. doi:10.1371/journal.ppat.1003558 23990782 PMC3749969

[200] Bayer NJ, Januliene D, Zocher G, Stehle T, Moeller A, Blaum BS. 2020. Structure of Merkel cell polyomavirus capsid and interaction with its glycosaminoglycan attachment receptor. J Virol 94:e01664-19. doi:10.1128/JVI.01664-19 32699083 PMC7527053

[201] Schowalter RM, Pastrana DV, Buck CB. 2011. Glycosaminoglycans and sialylated glycans sequentially facilitate Merkel cell polyomavirus infectious entry. PLoS Pathog 7:e1002161. doi:10.1371/journal.ppat.1002161 21829355 PMC3145800

[202] Neu U, Hengel H, Blaum BS, Schowalter RM, Macejak D, Gilbert M, Wakarchuk WW, Imamura A, Ando H, Kiso M, Arnberg N, Garcea RL, Peters T, Buck CB, Stehle T. 2012. Structures of Merkel cell polyomavirus VP1 complexes define a sialic acid binding site required for infection. PLoS Pathog 8:e1002738. doi:10.1371/journal.ppat.1002738 22910713 PMC3406085

[203] Becker M, Dominguez M, Greune L, Soria-Martinez L, Pfleiderer MM, Schowalter R, Buck CB, Blaum BS, Schmidt MA, Schelhaas M. 2019. Infectious entry of Merkel cell polyomavirus. J Virol 93:e02004-18. doi:10.1128/JVI.02004-18 30626687 PMC6401430

[204] Liu W, Yang R, Payne AS, Schowalter RM, Spurgeon ME, Lambert PF, Xu X, Buck CB, You J. 2016. Identifying the target cells and mechanisms of Merkel cell polyomavirus infection. Cell Host Microbe 19:775–787. doi:10.1016/j.chom.2016.04.024 27212661 PMC4900903

[205] Liu W, Krump NA, MacDonald M, You J. 2018. Merkel cell polyomavirus infection of animal dermal fibroblasts. J Virol 92:e01610-17. doi:10.1128/JVI.01610-17 29167345 PMC5790942

[206] Liu W, Krump NA, Buck CB, You J. 2019. Merkel cell polyomavirus infection and detection. J Vis Exp. doi:10.3791/58950 PMC6656558 30799855

[207] Shuda M, Feng H, Kwun HJ, Rosen ST, Gjoerup O, Moore PS, Chang Y. 2008. T antigen mutations are a human tumor-specific signature for Merkel cell polyomavirus. Proc Natl Acad Sci USA 105:16272–16277. doi:10.1073/pnas.0806526105 18812503 PMC2551627

[208] Passerini S, Prezioso C, Babini G, Ferlosio A, Cosio T, Campione E, Moens U, Ciotti M, Pietropaolo V. 2023. Detection of Merkel Cell Polyomavirus (MCPyV) DNA and transcripts in Merkel Cell Carcinoma (MCC). Pathogens 12:894. doi:10.3390/pathogens12070894 37513741 PMC10385104

[209] Sihto H, Kukko H, Koljonen V, Sankila R, Böhling T, Joensuu H. 2011. Merkel cell polyomavirus infection, large T antigen, retinoblastoma protein and outcome in Merkel cell carcinoma. Clin Cancer Res 17:4806–4813. doi:10.1158/1078-0432.CCR-10-3363 21642382

[210] Verhaegen ME, Mangelberger D, Harms PW, Vozheiko TD, Weick JW, Wilbert DM, Saunders TL, Ermilov AN, Bichakjian CK, Johnson TM, Imperiale MJ, Dlugosz AA. 2015. Merkel cell polyomavirus small T antigen is oncogenic in transgenic mice. J Invest Dermatol 135:1415–1424. doi:10.1038/jid.2014.446 25313532 PMC4397111

[211] Dye KN, Welcker M, Clurman BE, Roman A, Galloway DA. 2019. Merkel cell polyomavirus Tumor antigens expressed in Merkel cell carcinoma function independently of the ubiquitin ligases Fbw7 and β-TrCP. PLoS Pathog 15:e1007543. doi:10.1371/journal.ppat.1007543 30689667 PMC6366716

[212] Sergi MC, Lauricella E, Porta C, Tucci M, Cives M. 2023. An update on Merkel cell carcinoma. Biochim Biophys Acta Rev Cancer 1878:188880. doi:10.1016/j.bbcan.2023.188880 36914034

[213] Martel-Jantin C, Filippone C, Cassar O, Peter M, Tomasic G, Vielh P, Brière J, Petrella T, Aubriot-Lorton MH, Mortier L, Jouvion G, Sastre-Garau X, Robert C, Gessain A. 2012. Genetic variability and integration of Merkel cell polyomavirus in Merkel cell carcinoma. Virology (Auckl) 426:134–142. doi:10.1016/j.virol.2012.01.018 22342276

[214] Starrett GJ, Thakuria M, Chen T, Marcelus C, Cheng J, Nomburg J, Thorner AR, Slevin MK, Powers W, Burns RT, Perry C, Piris A, Kuo FC, Rabinowits G, Giobbie-Hurder A, MacConaill LE, DeCaprio JA. 2020. Clinical and molecular characterization of virus-positive and virus-negative Merkel cell carcinoma. Genome Med 12:30. doi:10.1186/s13073-020-00727-4 32188490 PMC7081548

[215] Czech-Sioli M, Günther T, Therre M, Spohn M, Indenbirken D, Theiss J, Riethdorf S, Qi M, Alawi M, Wülbeck C, Fernandez-Cuesta I, Esmek F, Becker JC, Grundhoff A, Fischer N. 2020. High-resolution analysis of Merkel cell polyomavirus in Merkel cell carcinoma reveals distinct integration patterns and suggests NHEJ and MMBIR as underlying mechanisms. PLoS Pathog 16:e1008562. doi:10.1371/journal.ppat.1008562 32833988 PMC7470373

[216] Starrett GJ, Marcelus C, Cantalupo PG, Katz JP, Cheng J, Akagi K, Thakuria M, Rabinowits G, Wang LC, Symer DE, Pipas JM, Harris RS, DeCaprio JA. 2017. Merkel cell polyomavirus exhibits dominant control of the tumor genome and transcriptome in virus-associated merkel cell carcinoma. mBio 8:e02079-16. doi:10.1128/mBio.02079-16 PMC5210499 28049147

[217] Laude HC, Jonchère B, Maubec E, Carlotti A, Marinho E, Couturaud B, Peter M, Sastre-Garau X, Avril M-F, Dupin N, Rozenberg F. 2010. Distinct merkel cell polyomavirus molecular features in tumour and non tumour specimens from patients with merkel cell carcinoma. PLoS Pathog 6:e1001076. doi:10.1371/journal.ppat.1001076 20865165 PMC2928786

[218] Nirenberg A, Steinman H, Dixon J, Dixon A. 2020. Merkel cell carcinoma update: the case for two tumours. J Eur Acad Dermatol Venereol 34:1425–1431. doi:10.1111/jdv.16158 31855292

[219] Sundqvist BZ, Kilpinen SK, Böhling TO, Koljonen VSK, Sihto HJ. 2023. Transcriptomic analyses reveal three distinct molecular subgroups of Merkel cell carcinoma with differing prognoses. Int J Cancer 152:2099–2108. doi:10.1002/ijc.34425 36620996

[220] Bill CA, Summers J. 2004. Genomic DNA double-strand breaks are targets for hepadnaviral DNA integration. Proc Natl Acad Sci USA 101:11135–11140. doi:10.1073/pnas.0403925101 15258290 PMC503752

[221] Yang L, Ye S, Zhao X, Ji L, Zhang Y, Zhou P, Sun J, Guan Y, Han Y, Ni C, Hu X, Liu W, Wang H, Zhou B, Huang J. 2018. Molecular characterization of HBV DNA integration in patients with hepatitis and hepatocellular carcinoma. J Cancer 9:3225–3235. doi:10.7150/jca.26052 30271481 PMC6160693

[222] Li X, Zhang J, Yang Z, Kang J, Jiang S, Zhang T, Chen T, Li M, Lv Q, Chen X, McCrae MA, Zhuang H, Lu F. 2014. The function of targeted host genes determines the oncogenicity of HBV integration in hepatocellular carcinoma. J Hepatol 60:975–984. doi:10.1016/j.jhep.2013.12.014 24362074

[223] Tian R, Wang Y, Li W, Cui Z, Pan T, Jin Z, Huang Z, Li L, Lang B, Wu J, Xie H, Lu Y, Tian X, Hu Z. 2022. Genome-wide virus-integration analysis reveals a common insertional mechanism of HPV, HBV and EBV. Clin Transl Med 12:e971. doi:10.1002/ctm2.971 35968887 PMC9376973

[224] Groves IJ, Coleman N. 2018. Human papillomavirus genome integration in squamous carcinogenesis: what have next-generation sequencing studies taught us? J Pathol 245:9–18. doi:10.1002/path.5058 29443391

[225] Porter VL, Marra MA. 2022. The drivers, mechanisms, and consequences of genome instability in HPV-driven cancers. Cancers (Basel) 14:4623. doi:10.3390/cancers14194623 36230545 PMC9564061

[226] Akagi K, Symer DE, Mahmoud M, Jiang B, Goodwin S, Wangsa D, Li Z, Xiao W, Dunn JD, Ried T, Coombes KR, Sedlazeck FJ, Gillison ML. 2023. Intratumoral heterogeneity and clonal evolution induced by HPV integration. Cancer Discov 13:910–927. doi:10.1158/2159-8290.CD-22-0900 36715691 PMC10070172

[227] Ceccaldi R, Liu JC, Amunugama R, Hajdu I, Primack B, Petalcorin MIR, O’Connor KW, Konstantinopoulos PA, Elledge SJ, Boulton SJ, Yusufzai T, D’Andrea AD. 2015. Homologous-recombination-deficient tumours are dependent on Polθ-mediated repair. Nature 518:258–262. doi:10.1038/nature14184 25642963 PMC4415602

[228] Leeman JE, Li Y, Bell A, Hussain SS, Majumdar R, Rong-Mullins X, Blecua P, Damerla R, Narang H, Ravindran PT, Lee NY, Riaz N, Powell SN, Higginson DS. 2019. Human papillomavirus 16 promotes microhomology-mediated end-joining. Proc Natl Acad Sci USA 116:21573–21579. doi:10.1073/pnas.1906120116 31591214 PMC6815166

[229] Chakraborty PR, Ruiz-Opazo N, Shouval D, Shafritz DA. 1980. Identification of integrated hepatitis B virus DNA and expression of viral RNA in an HBsAg-producing human hepatocellular carcinoma cell line. Nature 286:531–533. doi:10.1038/286531a0 6250073

[230] Lace MJ, Anson JR, Klussmann JP, Wang DH, Smith EM, Haugen TH, Turek LP. 2011. Human papillomavirus type 16 (HPV-16) genomes integrated in head and neck cancers and in HPV-16-immortalized human keratinocyte clones express chimeric virus-cell mRNAs similar to those found in cervical cancers. J Virol 85:1645–1654. doi:10.1128/JVI.02093-10 21123375 PMC3028875

[231] Gardella T, Medveczky P, Sairenji T, Mulder C. 1984. Detection of circular and linear herpesvirus DNA molecules in mammalian cells by gel electrophoresis. J Virol 50:248–254. doi:10.1128/jvi.50.1.248-254.1984 6321792 PMC255605

[232] Chang Y, Cheng S-D, Tsai C-H. 2002. Chromosomal integration of Epstein - Barr virus genomes in nasopharyngeal carcinoma cells. Head Neck 24:143–150. doi:10.1002/hed.10039 11891944

[233] Whitehouse A. 2011. Gardella gel analysis to detect Herpesvirus saimiri episomal DNA. Cold Spring Harb Protoc 2011:1524–1526. doi:10.1101/pdb.prot066969 22135664

[234] Begum S, Cao D, Gillison M, Zahurak M, Westra WH. 2005. Tissue distribution of human papillomavirus 16 DNA integration in patients with tonsillar carcinoma. Clin Cancer Res 11:5694–5699. doi:10.1158/1078-0432.CCR-05-0587 16115905

[235] Brooks EG, Evans MF, Adamson C-C, Peng Z, Rajendran V, Laucirica R, Cooper K. 2012. In situ hybridization signal patterns in recurrent laryngeal squamous papillomas indicate that HPV integration occurs at an early stage. Head Neck Pathol 6:32–37. doi:10.1007/s12105-011-0308-5 22052184 PMC3311939

[236] Xiong J, Cheng J, Shen H, Ren C, Wang L, Gao C, Zhu T, Li X, Ding W, Zhu D, Wang H. 2021. Detection of HPV and human chromosome sites by dual-color fluorescence in situ hybridization reveals recurrent HPV integration sites and heterogeneity in cervical cancer. Front Oncol 11:734758. doi:10.3389/fonc.2021.734758 34676167 PMC8523950

[237] Tokino T, Matsubara K. 1991. Chromosomal sites for hepatitis B virus integration in human hepatocellular carcinoma. J Virol 65:6761–6764. doi:10.1128/JVI.65.12.6761-6764.1991 1682510 PMC250761

[238] Redmond CJ, Fu H, Aladjem MI, McBride AA. 2018. Human papillomavirus integration: analysis by molecular combing and fiber-FISH. Curr Protoc Microbiol 51:e61. doi:10.1002/cpmc.61 30129235 PMC6209533

[239] Takada S, Gotoh Y, Hayashi S, Yoshida M, Koike K. 1990. Structural rearrangement of integrated hepatitis B virus DNA as well as cellular flanking DNA is present in chronically infected hepatic tissues. J Virol 64:822–828. doi:10.1128/JVI.64.2.822-828.1990 2296084 PMC249177

[240] Minami M, Poussin K, Bréchot C, Paterlini P. 1995. A novel PCR technique UsingAlu-specific primers to identify unknown flanking sequences from the human genome. Genomics 29:403–408. doi:10.1006/geno.1995.9004 8666388

[241] Tu T, Jilbert AR. 2017. Detection of hepatocyte clones containing integrated hepatitis B virus DNA using inverse nested PCR. Methods Mol Biol 1540:97–118. doi:10.1007/978-1-4939-6700-1_9 27975311

[242] Luft F, Klaes R, Nees M, Dürst M, Heilmann V, Melsheimer P, von Knebel Doeberitz M. 2001. Detection of integrated papillomavirus sequences by ligation-mediated PCR (DIPS-PCR) and molecular characterization in cervical cancer cells. Int J Cancer 92:9–17. doi:10.1002/1097-0215(200102)9999:9999<::AID-IJC1144>3.0.CO;2-L 11279600

[243] Matovina M, Sabol I, Grubišić G, Gašperov NM, Grce M. 2009. Identification of human papillomavirus type 16 integration sites in high-grade precancerous cervical lesions. Gynecol Oncol 113:120–127. doi:10.1016/j.ygyno.2008.12.004 19157528

[244] Schrama D, Sarosi E-M, Adam C, Ritter C, Kaemmerer U, Klopocki E, König E-M, Utikal J, Becker JC, Houben R. 2019. Characterization of six Merkel cell polyomavirus-positive Merkel cell carcinoma cell lines: integration pattern suggest that large T antigen truncating events occur before or during integration. Int J Cancer 145:1020–1032. doi:10.1002/ijc.32280 30873613

[245] Klaes R, Woerner SM, Ridder R, Wentzensen N, Duerst M, Schneider A, Lotz B, Melsheimer P, von Knebel Doeberitz M. 1999. Detection of high-risk cervical intraepithelial neoplasia and cervical cancer by amplification of transcripts derived from integrated papillomavirus oncogenes. Cancer Res 59:6132–6136. 10626803

[246] Ziegert C, Wentzensen N, Vinokurova S, Kisseljov F, Einenkel J, Hoeckel M, von Knebel Doeberitz M. 2003. A comprehensive analysis of HPV integration loci in anogenital lesions combining transcript and genome-based amplification techniques. Oncogene 22:3977–3984. doi:10.1038/sj.onc.1206629 12813471

[247] Kim S-H, Koo B-S, Kang S, Park K, Kim H, Lee KR, Lee MJ, Kim JM, Choi EC, Cho NH. 2007. HPV integration begins in the tonsillar crypt and leads to the alteration of p16, EGFR and c-myc during tumor formation. Int J Cancer 120:1418–1425. doi:10.1002/ijc.22464 17205528

[248] Cricca M, Morselli-Labate AM, Venturoli S, Ambretti S, Gentilomi GA, Gallinella G, Costa S, Musiani M, Zerbini M. 2007. Viral DNA load, physical status and E2/E6 ratio as markers to grade HPV16 positive women for high-grade cervical lesions. Gynecol Oncol 106:549–557. doi:10.1016/j.ygyno.2007.05.004 17568661

[249] Deng Z, Hasegawa M, Kiyuna A, Matayoshi S, Uehara T, Agena S, Yamashita Y, Ogawa K, Maeda H, Suzuki M. 2013. Viral load, physical status, and E6/E7 mRNA expression of human papillomavirus in head and neck squamous cell carcinoma. Head Neck 35:800–808. doi:10.1002/hed.23034 22791649

[250] Wang Q, Jia P, Zhao Z. 2013. VirusFinder: software for efficient and accurate detection of viruses and their integration sites in host genomes through next generation sequencing data. PLoS One 8:e64465. doi:10.1371/journal.pone.0064465 23717618 PMC3663743

[251] Fujimoto A, Furuta M, Totoki Y, Tsunoda T, Kato M, Shiraishi Y, Tanaka H, Taniguchi H, Kawakami Y, Ueno M, et al.. 2016. Whole-genome mutational landscape and characterization of noncoding and structural mutations in liver cancer. Nat Genet 48:500–509. doi:10.1038/ng.3547 27064257

[252] Wang A, Wu L, Lin J, Han L, Bian J, Wu Y, Robson SC, Xue L, Ge Y, Sang X, Wang W, Zhao H. 2018. Whole-exome sequencing reveals the origin and evolution of hepato-cholangiocarcinoma. Nat Commun 9:894. doi:10.1038/s41467-018-03276-y 29497050 PMC5832792

[253] Svicher V, Salpini R, Piermatteo L, Carioti L, Battisti A, Colagrossi L, Scutari R, Surdo M, Cacciafesta V, Nuccitelli A, Hansi N, Ceccherini Silberstein F, Perno CF, Gill US, Kennedy PTF. 2021. Whole exome HBV DNA integration is independent of the intrahepatic HBV reservoir in HBeAg-negative chronic hepatitis B. Gut 70:2337–2348. doi:10.1136/gutjnl-2020-323300 33402415 PMC8588301

[254] Tuna M, Amos CI. 2017. Next generation sequencing and its applications in HPV-associated cancers. Oncotarget 8:8877–8889. doi:10.18632/oncotarget.12830 27784002 PMC5352450

[255] Fukano K, Wakae K, Nao N, Saito M, Tsubota A, Toyoshima T, Aizaki H, Iijima H, Matsudaira T, Kimura M, Watashi K, Sugiura W, Muramatsu M. 2023. A versatile method to profile hepatitis B virus DNA integration. Hepatol Commun 7:e0328. doi:10.1097/HC9.0000000000000328 38051537 PMC10697629

[256] Yang W, Liu Y, Dong R, Liu J, Lang J, Yang J, Wang W, Li J, Meng B, Tian G. 2020. Accurate detection of HPV integration sites in cervical cancer samples using the nanopore MinION sequencer without error correction. Front Genet 11:660. doi:10.3389/fgene.2020.00660 32714374 PMC7344299

[257] Andersen K, Holm K, Tranberg M, Pedersen CL, Bønløkke S, Steiniche T, Andersen B, Stougaard M. 2022. Targeted next generation sequencing for human papillomavirus genotyping in cervical liquid-based cytology samples. Cancers (Basel) 14:652. doi:10.3390/cancers14030652 35158920 PMC8833452

[258] Liang J, Cui Z, Wu C, Yu Y, Tian R, Xie H, Jin Z, Fan W, Xie W, Huang Z, Xu W, Zhu J, You Z, Guo X, Qiu X, Ye J, Lang B, Li M, Tan S, Hu Z. 2021. DeepEBV: a deep learning model to predict Epstein-Barr virus (EBV) integration sites. Bioinformatics 37:3405–3411. doi:10.1093/bioinformatics/btab388 34009299

[259] Valmary-Degano S, Jacquin E, Prétet J-L, Monnien F, Girardo B, Arbez-Gindre F, Joly M, Bosset J-F, Kantelip B, Mougin C. 2013. Signature patterns of human papillomavirus type 16 in invasive anal carcinoma. Hum Pathol 44:992–1002. doi:10.1016/j.humpath.2012.08.019 23266444

[260] Lang B, Dong D, Zhao T, Zhong R, Qin H, Cao C, Wang Y, Liu T, Liang W, Tian X, Yan Y, Hu Z. 2023. A cross-sectional study of human papillomavirus genotype distribution and integration status in penile cancer among Chinese population. Virology (Auckl) 584:53–57. doi:10.1016/j.virol.2023.04.013 37244055

[261] Rodig SJ, Cheng J, Wardzala J, DoRosario A, Scanlon JJ, Laga AC, Martinez-Fernandez A, Barletta JA, Bellizzi AM, Sadasivam S, Holloway DT, Cooper DJ, Kupper TS, Wang LC, DeCaprio JA. 2012. Improved detection suggests all Merkel cell carcinomas harbor Merkel polyomavirus. J Clin Invest 122:4645–4653. doi:10.1172/JCI64116 23114601 PMC3533549

[262] Lim MY, Dahlstrom KR, Sturgis EM, Li G. 2016. Human papillomavirus integration pattern and demographic, clinical, and survival characteristics of patients with oropharyngeal squamous cell carcinoma. Head Neck 38:1139–1144. doi:10.1002/hed.24429 27002307

